# Development of an Antigen Delivery Platform Using *Lactobacillus acidophilus* Decorated With Heterologous Proteins: A Sheep in Wolf’s Clothing Story

**DOI:** 10.3389/fmicb.2020.509380

**Published:** 2020-10-27

**Authors:** Paula J. Uriza, Cynthia Trautman, María M. Palomino, Joaquina Fina Martin, Sandra M. Ruzal, Mara S. Roset, Gabriel Briones

**Affiliations:** ^1^Instituto de Investigaciones Biotecnológicas, Universidad Nacional de San Martín, IIB-UNSAM (IIBIO-CONICET), Buenos Aires, Argentina; ^2^Facultad de Ciencias Exactas y Naturales, Departamento de Química Biológica, Universidad de Buenos Aires, Buenos Aires, Argentina; ^3^CONICET – Universidad de Buenos Aires, Instituto de Química Biológica de la Facultad de Ciencias Exactas y Naturales (IQUIBICEN), Buenos Aires, Argentina

**Keywords:** *Lactobacillus*, shiga toxigenic *Escherichia coli*, S-layer (fusion) proteins, oral vaccination, antigen delivery system

## Abstract

S-layers are bacterial structures present on the surface of several Gram-positive and Gram-negative bacteria that play a role in bacterial protection. In *Lactobacillus acidophilus* (*L. acidophilus* ATCC 4356), the S-layer is mainly composed of the protein SlpA. A tandem of two copies of the protein domain SLP-A (pfam: 03217) was identified at the C-terminal of SlpA, being this double SLP-A protein domain (in short dSLP-A) necessary and sufficient for the association of the protein to the *L. acidophilus* cell wall. A variety of proteins fused to the dSLP-A domain were able to spontaneously associate with high affinity to the cell wall of *L. acidophilus* and *Bacillus subtilis* var. natto, in a process that we termed decoration. Binding of dSLP-A-containing-proteins to *L. acidophilus* was stable at conditions that mimic the gastrointestinal transit in terms of pH, proteases, and bile salts. To evaluate if protein decoration of *L. acidophilus* can be adapted to generate an oral vaccine platform, a chimeric antigen derived from the bacterial pathogen Shiga-toxin-producing *Escherichia coli* (STEC) was constructed by fusing the sequences encoding the polypeptides EspA^36–192^, Intimin^653–953^, Tir^240–378^, and H7 flagellin^352–374^ (EITH7) to the dSLP-A domain (EITH7-dSLP-A). Recombinantly expressed EITH7-dSLP-A protein was affinity purified and combined with *L. acidophilus* cultures to allow the association of the chimeric antigen to the bacterial surface. EITH7-decorated *L. acidophilus* was orally administered to BALB/c mice and the induction of anti-EITH7 specific antibodies in sera and feces determined by ELISA. Mice presenting significantly higher anti-EITH7 antibodies titers were able to control more efficiently an experimental STEC infection than mice that received the non-decorated *L. acidophilus* carrier, indicating that antigen-decorated *L. acidophilus* can be adapted as a mucosal immunization delivery platform to elicit a protective immune response for vaccine purposes.

## Introduction

Vaccines have been the most successful medical therapy in Public Health preventing mortality and morbidity caused by several infectious diseases and saving millions of lives worldwide (Orenstein and Ahmed, 2017). Although most microbial pathogens enter the body through a particular mucosal barrier (gastrointestinal, respiratory, or genital mucosa) only a few vaccines using this route of administration have been approved, probably due to certain limitations in their protection performance (Lycke, 2012). It has been described that some oral vaccines performed poorly in developing countries compared with industrialized ones and this has been attributed to a syndrome named chronic environmental enteropathy (or tropical enteropathy). This syndrome is characterized by impairment in intestinal absorption that might lead to a reduction of bioavailability of zinc or vitamin A affecting the efficiency of adjuvant function and, therefore, impairing the induction of an effective immune response (Lycke, 2012). Another limitation related to oral administration of antigens is the natural proteolytic degradation that proteins suffer in the intestinal tract. Despite these limitations, oral immunization remains an ideal vaccine route to be explored, especially in global campaigns of vaccination; because its administration is simple (needleless administration) and non-medical trained personnel can perform it. Live vaccines based on attenuated microorganisms have been shown to be a very effective way to elicit a protective immunity as a consequence of the ability of the vaccine strain to replicate inside the host resembling the real infection, therefore, providing an effective antigenic stimulation that leads to the development of a protective memory immune response. The selection of attenuated microorganisms has been historically a serendipitous process based on the sequential replication of a pathogen either in laboratory *in vitro* conditions or within a non-natural host in order to isolate non-virulent strains (Plotkin, 2014; Minor, 2015). By this method, a few protective vaccines have been developed that have been widely used such as Oral Polio Virus (Sabin), BCG for tuberculosis, and the Yellow Fever vaccine YF17D (Galler et al., 1997; Plotkin, 2014). Despite a better performance of live-attenuated vaccines to induce individual and population protection, there are certain limitations such as the latent possibility of reversion to a full virulence or the risk that the residual virulence of a vaccine strain might be still dangerous for immune-compromised individuals. In addition, not all the microbial pathogens can be cultured *in vitro* and, in some cases, special biosafety facilities are also required, increasing production costs. To sort out these limitations, it has been proposed the use of vaccine strains as carriers for expressing heterologous antigens. Recently, a recombinant tetravalent vaccine against Dengue virus using the Yellow fever vaccine as a carrier was approved for humans, although, as a consequence of some complications that appeared during Phase III, the vaccine has had a limited use (Larson et al., 2019). In the case of vaccines based on genetically modified organisms (GMOs), they are subjected to strict regulations to allow its administration to animals or humans increasing production costs considerably (Seib et al., 2017).

Members of the *Lactobacillus* genus are considered as generally recognized as safe (GRAS) bacteria, since they are natural commensal bacteria that inhabit the gastrointestinal tract and genital mucosa and have been used for centuries in the preparation of fermented foods like yogurt, kefir, or sour cream. Given their mucosal localization, *Lactobacillus* species have been recently proposed as a recombinant carrier for oral vaccine development. For example, the human immunodeficiency virus (HIV) Gag, heat labile enterotoxin, rotavirus VP4, and the Helicobacter Adhesin Hp0410, have been expressed in *Lactobacillus* strains for vaccine purposes (Lecureux and Dean, 2018). However, these *Lactobacillus* strains are DNA-recombinant vaccines, which affect their GRAS status.

When residing in the gut, some *Lactobacillus* species interact with host cells exerting a strain-specific immunomodulatory effect, that contributes to its probiotic properties (Hymes et al., 2016; Johnson et al., 2016; Douillard and de Vos, 2019). This immune-modulation has been attributed to the recognition of different microbial-associated molecular patterns (MAMPs) mediated by the pattern recognition receptors (PRRs), expressed in immune and non-immune host cells (Abou-Saleh et al., 2016; Hymes et al., 2016; Douillard and de Vos, 2019). MAMPs-PRPs interaction triggers an inflammatory response, which is the main mechanism of action of adjuvants, such as the observed in the bacterium-like particles or Gram-positive enhancer matrixes used as antigen delivery platforms (Arce et al., 2019). In this report, we adapted *Lactobacillus acidophilus* as an oral vaccine platform to deliver heterologous antigens from a bacterial intestinal pathogen, the Shiga-toxin-producing *Escherichia coli* (STEC). STEC is a bacterial pathogen that causes hemorrhagic enteritis and the life-threatening hemolytic uremic syndrome (HUS) in humans (Goldwater, 2007). Immunization with STEC surface proteins (such as EspA, Tir, Intimin, or flagellin) induces an antibody humoral response that interferes with the effective STEC-host cell interaction. Based on this observation, two vaccine candidates have been developed for the prevention or reduction of STEC intestinal colonization in cattle (Potter et al., 2004; Babiuk et al., 2008). STEC deploys protein secretion machinery, the type three secretion system (T3SS), critical for host invasion and intestinal colonization. STEC T3SS is a “nanomachine” that the bacteria utilize to translocate effector proteins to the host cell cytosol to promote its tight association to the host cell surface. Initially, the effector protein Tir is “injected” by the STEC T3SS within the host cells and exposed on the host membrane, where it will be recognized by Intimin, a STEC outer membrane protein. When Tir interacts with Intimin, a host-signaling cascade is triggered that leads to the rearrangement of the intestinal epithelial cellular architecture, inducing the formation of actin “pedestals” beneath the attached bacteria. In addition, the T3SS effector protein EspA polymerizes forming a filamentous tip on the bacterial membrane which is essential for bacterial attachment and T3SS activity. Here, we constructed a STEC chimeric antigen composed by the peptides EspA^36–192^, Intimin^653–935^ (containing the Tir-binding domain), Tir^258–361^ (containing the Intimin-binding domain), and H7 flagellin^352–374^ (EITH7), which is protective against an *E. coli* O157:H7 challenge infection in the mouse model (Iannino et al., 2015), and fused in frame with the peptide SlpA^284–444^ of *L. acidophilus*. The resulting EITH7-SlpA^284–444^ was recombinantly expressed and mixed with a *L. acidophilus* culture to allow the external coating of the bacteria with the STEC antigen, in a process that we termed “decoration.”

The *L. acidophilus* S-layer is composed mainly of SlpA, although there are less-represented proteins such as SlpX (Palomino et al., 2016). After being secreted, the S-layer proteins can self-assemble forming a bi-dimensional symmetrical structure that completely covers the bacterial cell wall. In *L. acidophilus*, SlpA, in addition to its function as a protective coat for the bacteria, also participates in host cell adhesion (Ashida et al., 2011). It has been described that the S-layer can be removed from the bacterial surface by cation substitution solutions (LiCl) or hydrogen bond-breaking agents such as guanidine hydrochloride (GHCl) or urea (Sleytr et al., 2014). Interestingly, after dialysis, the isolated S-layer protein can associate back to the *Lactobacillus* cell wall or adhere to inert surfaces that can be adapted for biotechnological purposes (Sleytr et al., 2014; Fina Martin et al., 2019). SlpA encodes in its carboxy-terminal (SlpA^284–444^) a tandem of two copies of the protein domain denominated SLP-A (dSLP-A), a region that was shown to be necessary and sufficient for the association of SlpA to the bacterial cell wall (Fina Martin et al., 2019). Teichoic acid and wall teichoic acid present on the lactobacilli bacterial cell wall have been characterized as natural ligands for the dSLP-A domain (Fina Martin et al., 2019). The dSLP-A domain (pfam: 03217) is found only in *Lactobacillus* species, limited to S-layer forming lactobacilli of the *L. acidophilus* group, including *Lactobacillus helveticus*, *Lactobacillus crispatus*, *Lactobacillus gallinarum*, and *Lactobacillus amylovorus* (Avall-Jaaskelainen and Palva, 2005). This domain is also found in S-layer associated proteins (Johnson et al., 2013) related to probiotic activity, adhesion (Hymes et al., 2016), immunomodulation (Johnson et al., 2017), and cell division (Johnson et al., 2016).

We evaluated the efficiency of *L. acidophilus* decorated with STEC antigens as a vaccine platform. Mice were orally immunized with EITH7-decorated *L. acidophilus* to evaluate its ability to elicit a protective immune response against STEC. Our results suggest that the antigen decoration strategy of lactobacilli can be an interesting alternative as a vaccine platform.

## Materials and Methods

### Bacterial Strains, Plasmids, and Culture Conditions

Bacterial strains and plasmids used are summarized in Table 1. *Escherichia coli* strains were cultured at 37°C on Luria Bertani (LB) medium (Sigma, St. Louis, MO, United States) or Sorbitol MacConkey agar (SMAC; Britania, Argentina). *Lactobacillus acidophilus* ATCC 4356 was cultured at 37°C on DeMan, Ragosa, and Sharpe medium (MRS; Britania, Argentina). *Bacillus subtilis* var. natto (ATCC 15245) was cultured in LB or LB agar. When necessary, culture media were supplemented with antibiotics at the following concentrations: ampicillin 50 μg/ml, kanamycin 50 μg/ml, and nalidixic acid 20 μg/ml. All experiments involving STEC were conducted in a BSL3 facility.

**Table 1:** Bacterial strains and plasmids used in this study.

Strain or plasmid	Relevant phenotype or genotype^a^	Reference or resource
Strain		
*E. coli*		
XL1-Blue MRF´	Δ(*mcrA*)*183* Δ(*mcrCB-hsdSMR-mrr*)*173 endA1 supE44 thi-1 recA1* gyrA96 relA1 lac [*F'* proAB lacI^q^*ZΔ*M15 *Tn*10,Tet^r^]	Stratagene
DH5α	F^−^ φ80*lac*ZΔM15 Δ(*lac*ZYA-*arg*F) U169 *rec*A1 *end*A1 *hsd*R17(r_K_^−^, m_K_^+^) *pho*A *sup*E44λ- *thi*-1 *gyr*A96 *rel*A1	Invitrogen
BL21 Codon plus	[*omp*T *hsd*S(r_B_^–^ m_B_^–^) *dcm* + Tc^r^ *gal* λ (DE3) *end*A Hte Cm^r^]	Stratagen
EDL933	Reference strain of STEC O157:H7 (stx1 stx2 eae-γ)	ATCC 43895
*L. acidophilus*	Wild type strain	ATCC 4356
*B. subtilis*	Wild type strain var. natto	ATCC 15245
Plasmid		
pIDT-SlpA	603 bp fragment encoding last C-terminal 160 acids of SlpA protein from *Lactobacillus acidophillus* with a FLAG tag (DYKDDDDK)	IDT, United States
pGEX-2T	4.9-kb expression vector N-terminal GST tag, C-terminal FLAG-Tag, Amp^r^	GE Healthcare
pGEX-SlpA	~600 pb BamHI/EcoRI fragment containing the synthetic C-terminal domain for *Lactobacillus acidophilus* SlpA protein cloned into pGEX-2 T, Amp^r^	This study
pUC57- EITH_7_	~2-kb BamHI/XbaI fragment containing the synthetic genes for EspA, Intimin, Tir and flagellin H7 domain (EITH7) from EDL933 strain cloned into pUC57, Amp^r^	GenScript NJ, United States
pLC3	Modified form of the expression vector pET-28 d (Novagen) with a maltose binding protein (MBP)-coding region inserted into the multiple cloning site (MCS).	Savinov et al., 2012
pLC3-EITH_7_	1841 pb NdeI/EcoRI restriction fragment from pUC57-EITH7 cloned into pLC3 plasmid.	This study
pLC3-EITH_7_-SlpA	505 pb PCR product of SlpA was cloned downstream EITH7 sequence into pLC3-EITH_7_ plasmid using EcoRI/HindIII restriction sites.	This study
pET-22b-Omp19	The ORF of unlipidated Omp19 protein of *Brucella abortus* was cloned in the pET-22b (Novagen, Madison, WI) expression vector.	Ibañez et al., 2015
pLC3-Omp19-SlpA	489 pb of Omp19 PCR product were inserted into NdeI/SalI restriction sites of pLC3-EITH_7_-SlpA replacing EITH7 sequence and obtaining pLC3-Omp19-SlpA plasmid.	This study
pET-22b-FliC	1,512 bp corresponding to *Salmonella enterica* Serovar Typhimurium LT2 were cloned in NdeI-and XhoI of pET22b.	GenScript NJ, United States
pLC3-FliC-SlpA	FliC sequence was obtained by NdeI/XhoI digestion of pET-22b-FliC vector and cloned into NdeI/SalI restriction sites of pLC3-EITH7-SlpA replacing EITH_7_ sequence by FliC sequence.	This study
pTRCHisB-Gal8 pLC3-Gal8-SlpA	990 pb NheI/BglII fragment containing the synthetic gen for murine Gal8 cloned into pTRC, Amp^r^ ~990 pb of Gal8 product, obtained by PCR of pTRCHisB-Gal8, were inserted into NdeI/SalI restriction sites of pLC3-EITH7-SlpA replacing EITH7 sequence.	This study This study

a Amp^r^, ampicillin resistance; Nal^r^, nalidixic acid resistance; Cm, Chloramphenicol; Kan^r^, Kanamycin resistance.

### Cloning, Expression, and Purification of Recombinant Proteins

A DNA fragment encoding for the last 160 amino acids of SlpA (SlpA^284–444^) of *L. acidophilus* that contains a tandem of two SLP-A protein domains (dSLP-A) was synthesized by Integrated DNA Technologies, Inc. (IDT, United States). A synthetic gene encoding EspA^36–192^, Intimin^653–953^, Tir^240–378^, and flagellin H7domain (EITH7) from STEC EDL933 strain was synthesized by GenScript Company (United States; Iannino et al., 2015). The peptide (EAAAK)_4_ was intervened between individual proteins to promote their proper folding (Iannino et al., 2015). The FLAG epitope (DYKDDDDK) was fused to the C-terminal of EITH7 to monitor protein expression.

The DNA sequence encoding the *L. acidophilus* dSLP-A domain (SlpA^284–444^) was subcloned into pGEX-2T vector (GE-Healthcare; Table 1), and the resultant protein GST-dSLP-A was expressed and purified by Glutathione Sepharose Fast Flow (GE-Healthcare) affinity chromatography as described by manufacturer instruction. The encoding sequence of EITH7 was subcloned into pLC3 expression vector (Table 1) and afterward the dSLP-A encoding sequence was placed at the C-terminal as shown in Table 1. The resulting plasmid pLC3-EITH7-dSLP-A was transformed into *E. coli* BL21. Expression of EITH7-dSLP-A protein was induced by a final concentration of 0.25 mM of Isopropyl β-D-1-thiogalactopyranoside (Sigma-Aldrich) and purified by amylose affinity chromatography (New England BioLabs) as manufacturer instruction.

Sequences of *fliC* from *Salmonella enterica* serovar Typhimurium LT2, *Brucella abortus omp19*, and the mouse lectin *gal8* were cloned into pLC3-EITH7- dSLP-A, replacing EITH7 from the construction (Table 1). Resulting M-FliC-dSLP-A, M-Omp19-dSLP-A, and M-Gal8-dSLP-A proteins was recombinantly expressed as described above and purified at the same experimental conditions used for M-EITH7-dSLP-A. For amplification of the sequences of Gal8 and Omp19 by PCR, the following primers were used: pFwGal8 5'-tgaCATATGgctagcatgttgtccttaaa-3', pRvGal8, 5'-gtaGTCGACccagctccttacatccag-3', pFwOmp19 5'-tgaCATATGatgcagagctcccggctt-3', and pRvOmp19 5'-tgaGTCGACgcgcgacagcgtcacgg-3'.

### Generation of Antibodies

Eight-week-old BALB/c mice were immunized intraperitoneally with 10 μg of purified recombinant GST-dSLP-A protein or M-EITH7 protein using aluminum hydroxide (Imject Alum ThermoFisher) as an adjuvant and boosted at 2–4 weeks with 5 μg of each protein. A week after the last immunization, mice were bled, and sera were stored at −20°C for further use.

### SDS-PAGE and Western Blot Analysis

Protein samples were treated with cracking buffer (50 mM Tris-HCl pH 6.8, 2% wt/vol sodium dodecyl sulfate, 0.1% wt/vol bromophenol blue, 10% wt/vol glycerol, and 50 mM dithiothreitol) and incubated at 100°C for 5 min. Protein electrophoresis separation was performed at 120 V for 1 h on a 10% SDS-PAGE gel. Subsequently, proteins were transferred to a nitrocellulose membrane using a semi-dry electroblotting transfer unit (Bio-Rad, Hercules, CA, United States) for 45 min at 15 V. After 1 h blocking buffer [0.1% Tween 20 and 1% dry skim milk in phosphate-buffered saline (PBS) buffer], membranes were incubated for 1 h with the corresponding primary antibody. Membranes were washed four times during 5 min with washing buffer (0.1% Tween 20 in PBS buffer) and incubated for 1 h with a 1:20.000 blocking buffer dilution of IRDye fluorophore-labeled secondary antibodies (LI-COR, Lincoln, NE, United States). After washing four times, membranes were scanned using the Odyssey Imaging System (LI-COR).

### *Lactobacillus* Decoration

Overnight cultures of lactobacilli were harvested by centrifugation. After centrifugation, the bacterial pellet was washed three times with cold PBS. An equal number of lactobacilli (5 × 10^8^ CFU) were resuspended and incubated for 30 min with different concentrations of dSLP-A-fusion proteins in a final volume of 100 μl. Each fraction of decorated lactobacilli was washed three times with cold PBS. *L. acidophilus* was pre-treated for 15 min with a 5 M LiCl solution to remove the *wild type* SlpA. The same protocol was used also for decoration of *B. subtilis*.

### Studies of Binding Stability

For studies of binding stability, decorated bacteria were subjected to different conditions that mimic the gastrointestinal environment (pH, bile acids, and protease). After being collected by centrifugation, *L. acidophilus* was washed twice with PBS and processed for SDS-PAGE and western blot analysis. GST-dSLP-A decorated bacteria were incubated 30 min at pH2 (stomach-like conditions), 60 min at pH6.5 (proximal small bowel-like), 120 min at pH7.5 (mid-small bowel-like), and 240 min at pH7.5 (distal small bowel-like; Evans et al., 1988). For protease stability assay, a pancreatin (SIGMA-ALDRICH) concentration of 10 μg/ml was used. For bile acid stability were used different concentrations of bile acids (Britania; 4–40 mg/ml) at different incubation times (10, 15, 30, and 60 min; Pfeiler et al., 2007).

### Mouse Vaccination

Eight-week-old BALB/c mice (female) were orally immunized with 4 × 10^8^ UFC of lactobacilli or 4 × 10^8^ CFU of lactobacilli carrying 20 μg of M-EITH7-dSLP-A or with M-EITH7-dSLP-A alone in a three doses scheme (0, 2, and 4 weeks). Prior oral immunization mice were fasted for 6 h and food was placed back 1 h after oral immunization.

### Challenge of Vaccinated Mice With Experimental Infection of STEC (*Escherichia coli* O157:H7)

Ten days after the last immunization, mice were challenged by intragastric inoculation of 10^10^ CFU of STEC in 100 μl of PBS. Previously, food was removed from mouse cages 6 h before the oral infection challenge. Bacterial fecal shedding was monitored daily for over 25 days. Briefly, mouse fecal pellets were collected, suspended in 500 μl of PBS, serially diluted, and plated onto SMAC agar with 20 μg/ml of nalidixic acid. Culture plates were incubated at 37°C for 24 h, and colony-forming units (CFUs) were determined.

### EITH7-Specific Antibody Determination

The 96-well plate was coated with purified M-EITH7 protein (Table 1; 125 ng/well) in carbonate-bicarbonate buffer pH 9.6 for the iELISA test. To determine fecal antibodies, feces were resuspended in buffer PBS containing 1% of BSA (Sigma, St. Louis, MO, United States), 25 mM of EDTA, 2 mM of phenylmethylsulfonyl fluoride (PMSF; Sigma, St. Louis, MO, Unites States). The suspension was clarified by centrifugation and its supernatant diluted (1:2) and then used for an ELISA test during the same day. Sera samples were collected and stored at −20°C until use. Sera were 1:200 diluted in the blocking buffer (0.1% Tween 20 in PBS buffer). To reveal antibody presence, HRP-conjugated anti-mouse IgA (1:500) or HRP-anti-mouse-IgG (1:1000) were added and incubated for 1 h at 25°C. HRP activity was determined with 3, 3',5,5' Tetramethylbenzidine (Sigma), and optical density was measured at 450 nm (OD450). A positive serum was generated against the EITH7 antigen by ip immunization that was included in all the ELISA test plates as an internal reference value.

### Immunofluorescence Microscopy

*Lactobacillus acidophilus* or *B. subtilis* previously decorated with dSLP-A-tagged proteins were incubated during 30 min on coverslips pretreated with poly-L-Lysine (Sigma, St. Louis, MO, United States) to allow the bacterial adhesion. After washing twice with PBS, samples were fixed with PFA (4% in PBS) for 15 min at room temperature. After fixation, coverslips were washed twice with PBS, and the remaining PFA was quenched by incubation with 50 mM NH4Cl (Sigma, St. Louis, MO, United States) for 1 h or overnight. Coverslips were washed with PBS and blocked during 1 h with 10% Bovine Serum Albumin (Sigma, St. Louis, MO, United States) in PBS. Later, covers were incubated 1 h with anti-EITH7 mouse sera diluted (1:500) in blocking buffer, washed with PBS, and incubated 1 h more with an ALEXA488- anti-IgG conjugated (ThermoFisher), diluted (1:10000) in blocking buffer. Finally, samples were examined with a confocal laser-scanning microscope Olympus FV1000 using a PlanApo N (60 × 1.42 NA) oil objective.

### Statistical Analysis

One-way ANOVA with Bonferroni *post hoc* test and Student’s *t*-test (GraphPad Software) was used for statistical analysis.

### Ethics Statement

Experimental procedure of this study (permit number 15/2018) was approved by the Committee on the Ethics of Animal Experiments of the Universidad Nacional de San Martín (UNSAM), under the recommendations for animal experimentation (Helsinki Declaration and its amendments, Amsterdam Protocol of welfare and animal protection and National Institutes of Health, United States NIH, guidelines: Guide for the Care and Use of Laboratory Animals).

## Results

### Proteins Fused in Frame With the dSLP-A Protein Domain Can Associate With the Surface of *L. acidophilus*

**Figure 1:**
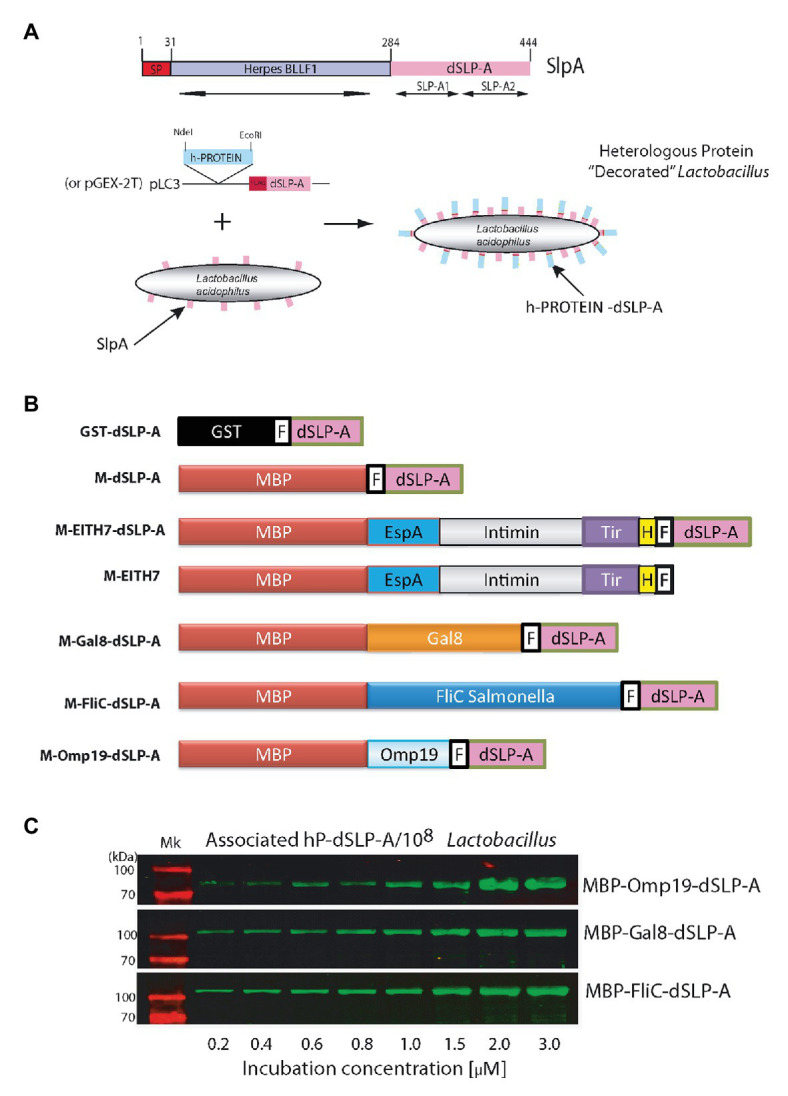
**(A)** The scheme shows all the protein domains detected in the sequence of the protein *L. acidophilus* SlpA predicted by the NCBI BLAST program. The binding domain named dSLP-A is formed by a tandem of two copies of the SLP-A domain (pfam03217): SLP-A1 and SLP-A2. SlpA^1–31^ encodes for a secretion signal sequence predicted by SignalP-5.0 (SP). In SlpA ^31–284^ region, a Herpes BLLF1 domain was detected (pfam05109). A cartoon showing the decoration process is presented which includes the expression vector used for tagging different heterologous proteins (h-PROTEIN) with the dSLP-A domain. **(B)** Different constructions used in this report. **(C)** Western blot analysis shows the increasing association of dSLP-A-tagged proteins to the *Lactobacillus* cell wall that correlates with the increasing concentration of these proteins in the incubation solution. TransGen Biotech Pre-stained markers blue-Plus Pre-stained markers 120, 100, 70, 50, 40, 30, 25, and 14 kDa. GST, glutathione S-transferase; MBP, maltose-binding protein; F, FLAG epitope; H, *Escherichia coli* H7 epitope.

To analyze the presence of conserved protein domains within the SlpA amino acid sequence, a BLAST search was performed. As shown in Figure 1A, two copies of a 60-amino acids protein domain denominated SLP-A (pfam03217; Figure 1A; Boot et al., 1995) were identified within de carboxy- terminal of SlpA. Both copies of the SLP-A domain (dSLP-A) are required for an efficient cell wall binding of SlpA to the surface of *L. acidophilus* (Fina Martin et al., 2019). In addition, as shown in Figure 1A, the amino-terminal of SlpA (SlpA^1–31^) encodes a predicted signal peptide (analyzed by a deep neural network-based method SignalP-5.0) and the peptide region SlpA^31–284^ for a Herpes BLLF1 protein domain (pfam05109), a protein region that has been shown to be required for self-assembly of SlpA on the bacterial surface to form the S-layer (Smit et al., 2002). As shown in the scheme presented in Figure 1A, an expression vector was constructed for fusing in frame any heterologous proteins (h-proteins) of interest with the dSLP-A domain. Also, the sequence of the FLAG epitope was inserted between the heterologous protein of interest and the dSLP-A domain as a reporter tag. As shown in Figure 1B and Table 1, two different plasmids backbones were used for cloning proteins of interest, fused to the dSLP-A domain: the pLC3 (Savinov et al., 2012) and the plasmid pGEX-2T that allows the fusion of polypeptides with the maltose-binding protein (MBP) or the glutathione S-tranferase (GST), respectively, both for affinity purification purposes. Consequently, a set of dSLP-A-fused proteins were constructed and were used here (Figure 1B). As shown in Figure 1C, three different examples of heterologous proteins such as the *Brucella* protease inhibitor Omp19 (Ibañez et al., 2015), the *Salmonella* flagellin FliC (Mcclelland et al., 2001), and the mouse lectin GAL8 (Tribulatti et al., 2007) fused to the dSLP-A domain were able to efficiently bind to 10^8^ CFU of *L. acidophilus*.

### Adhesion of GST-dSLP-A Reporter Protein to the *L. acidophilus* Cell Wall Is Stable at Conditions That Mimic the Traffic Across the Intestinal Tract

**Figure 2:**
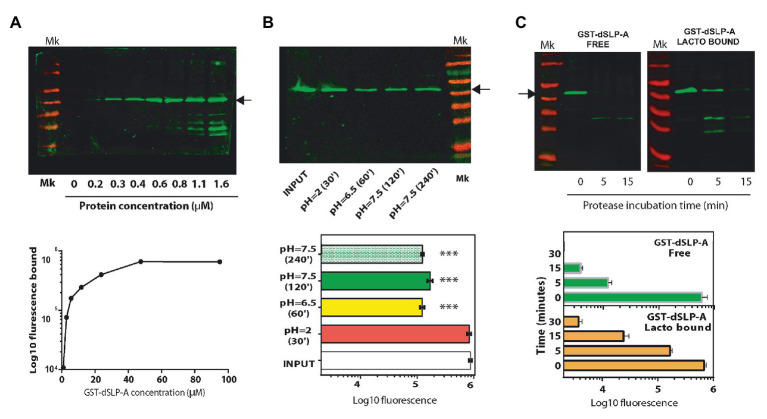
**(A)** Western blot analysis revealed with the Odyssey system (up panel), and its quantification (down panel) using the Image Studio software showing the association of the protein GST-dSLP-A to the surface of 10^8^ cells of *L. acidophilus*. Increasing concentrations of GST-dSLP-A (at μM concentration) correlates with the increasing amount of this protein associated with the bacterial cell wall that follows a typical saturation curve. **(B)** Western blot showing the amount of retained GST-dSLP-A on the bacterial cell wall after the incubation for 30 min at pH2 (stomach-like conditions), 60 min at pH6.5 (proximal small bowel-like), 120 min at pH7.5 (mid-small bowel-like), and 240 min at pH7.5 (distal small bowel-like) compared with the control (INPUT; up panel). Quantification is shown in the down panel. One way ANOVA test and Bonferroni *post hoc* test were performed to analyze the statistical significance of these results (^***^p < 0.001). **(C)** Western blot analysis comparing the speed of degradation of GST-dSLP-A bound to *L. acidophilus* or GST-dSLP-A free in solution incubated with pancreatin (free vs. bound). Arrow indicates the migration of GST-dSLP-A (47.2 kDa). Thermo Scientific Pre-stained markers 180, 130, 100, 70, 55, 40, 35, 25, 15, and 10 kDa. Results shown are representative of two independent experiments.

As described in Table 1, the sequence of the dSLP-A domain was inserted in pGEX-2T to construct the reporter protein GST-dSLP-A (Figure 1B). *Lactobacillus acidophilus* decorated with GST-dSLP-A were subjected to environmental conditions that bacteria encounter during the transit through the intestinal tract (pH variations, bile salts, or degradation by intestinal proteases) to evaluate the stability of dSLP-A binding in these conditions (Figure 2).

As shown in Figure 2A, GST-dSLP-A coating of *L. acidophilus* follows a saturation curve reaching a plateau at micromolar concentrations. The stability of the GST-dSLP-A association with *L. acidophilus* wall was tested in the environmental conditions mimicking different portions of the human intestinal tract (stomach, duodenum, jejunum, and ileum) in terms of pH and time of transit through the intestinal tract (Evans et al., 1988). As shown in Figure 2B, GST-dSLP-A (INPUT) binding to *L. acidophilus* was stable for 30 min at pH 2 and 37°C, a condition that resembles the stomach-like environment. In addition, the stability of GST-dSLP-A bacterial association at proximal small bowel-like conditions (1 h, pH 6.5–37°C), mid-small bowel-like conditions (2 h, pH 7.5–37°C), and distal small bowel-like conditions (4 h, pH 7.5–37°C) was partially affected compared against the input control (*p* < 0.001; Figure 2B). No significant difference was detected between treatments at pH 6.5/37°C, 2 h or 4 h at pH7.5/37°C. Interestingly, GST-dSLP-A association with *L. acidophilus* was not affected by increasing concentration of bile salts even when the concentration was five times higher than physiological concentrations (Supplementary Figure S1; Pfeiler et al., 2007).

As shown in Figure 2C, binding of GST-dSLP-A to *L. acidophilus* protected the degradation of this protein when incubated with the digestive enzymes cocktail named Pancreatin (containing trypsin, chymotrypsin, pepsin, pancreatin, and elastase) compared with an equivalent amount of non-associated GST-dSLP-A. Free GST-dSLP-A was degraded close to 10 times faster than GST-dSLP-A bound to *L. acidophilus*.

### The Chimeric Antigen EITH7-dSLP-A Binds to *L. acidophilus* Surface Following A Saturation Curve

**Figure 3:**
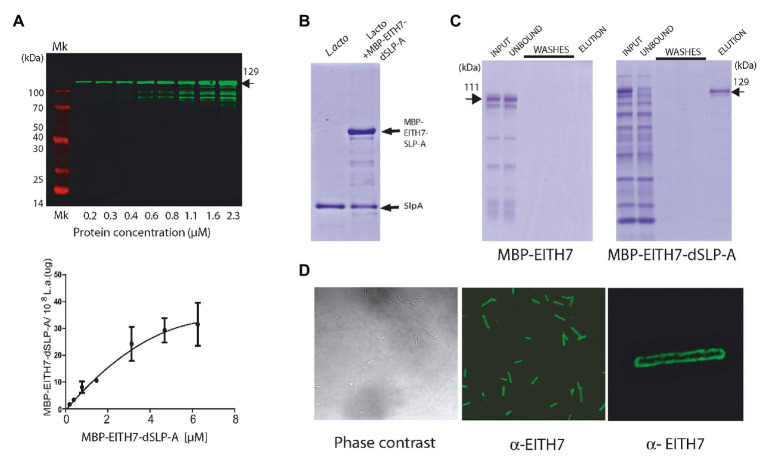
**(A)** Western blot (up panel) and its quantification (down panel) showing the association of the MBP-EITH7-dSLP-A to 10^8^ cells of *L. acidophilus*. Incubation with increasing concentration of MBP-EITH7-dSLP-A correlates with the increment in the association of this protein to the bacterial cell wall that follows a typical saturation curve. As described, by comparison with a standard curve that correlates micrograms of proteins with the log_10_ of the fluorescence is possible to convert fluorescence to micrograms of MBP-EITH7-dSLP-A bound to *Lactobacillus* (down panel). **(B)** SDS-PAGE analysis was performed to evaluate the binding of MBP-EITH7-dSLP-A to *Lactobacillus*. Purified MBP-EITH7-dSLP-A was incubated with 10^8^ bacteria for 30 min at 4°C and afterward centrifuged to separate bacterial pellet from the protein input solution. After two washes the pellet was subjected to 5 M LiCl to elute bacterium-associated proteins. **(C)** To control binding specificity of MBP-EITH7-dSLAP to *Lactobacillus*, proteins MBP-EITH7 (as a negative control) and MBP-EITH7-dSLP-A were incubated with 10^8^ bacteria for 30 min at 37°C, centrifuged, and the bacterial pellet were washed twice and resuspended in 5 M LiCl to elute protein associated to *Lactobacillus*. **(D)** Immune-fluorescence using an anti-EITH7 antibody. Confocal microscopy shows the homogenous distribution of MBP-EITH7 on the *L. acidophilus* surface after decoration. TransGen Biotech Pre-stained markers blue-Plus Pre-stained markers 120, 100, 70, 50, 40, 30, 25, and 14 kDa.

To evaluate the possibility of using *L. acidophilus* as an antigen delivery system, the chimeric antigen EspA^36–192^-Intimin^653–953^-Tir^240–378^ H7-flagellin^352–374^ (EITH7; from *E. coli* O157:H7 EDL933) was constructed by gene synthesis as described in the section Materials and Methods, fused in frame with the dSLP-A domain and subcloned into the pLC3 expression vector (MBP-EspA^36–192^-Intimin^653–953^-Tir^240–378^- H7-flagellin^352–374^ SlpA^284–444^ or M-EITH7-dSLP-A; Figure 1B). As shown in Figure 3A, the protein M-EITH7-dSLP-A strongly associated with the cell wall of *L. acidophilus* with a similar saturation curve as the one observed with GST-dSLP-A (Figure 2A).

As described in the section Material and Methods, we estimated that 10^8^ cells of *L. acidophilus* were able to load up to 30 μg of protein M-EITH7-dSLP-A (Figure 3A, lower panel). Interestingly, to remove the recombinant S-layer attached to *L. acidophilus* cell wall formed by M-EITH7-dSLP-A, it was necessary to treat the antigen decorated bacteria with chaotropic solutions (5 M LiCl), with the same stringent conditions required for removing the *wild type* S-layer (Figure 3B).

To determine the strict requirement of dSLP-A domain for the binding specificity of M-EITH7-dSLP-A to *L. acidophilus*, cultures of these bacteria were incubated with M-EITH7 (without dSLP-A, see Figure 1B) or M-EITH7-dSLP-A (Figure 3C). Figure 3C shows that incubation with *L. acidophilus* retained proteins with high specificity from a protein lysate input only when dSLP-A was present in the construct (see input vs. unbound fraction). Accordingly, elution with 5 M LiCl only released proteins in the case of M-EITH7-dSLP-A indicating that the binding was dependent on the dSLP-A domain. When observed by immune-fluorescence confocal microscopy, it was possible to determine that M-EITH7-dSLP-A completely coated the surface of the bacteria *L. acidophilus* with a homogeneous distribution (Figure 3D).

**Figure 4:**
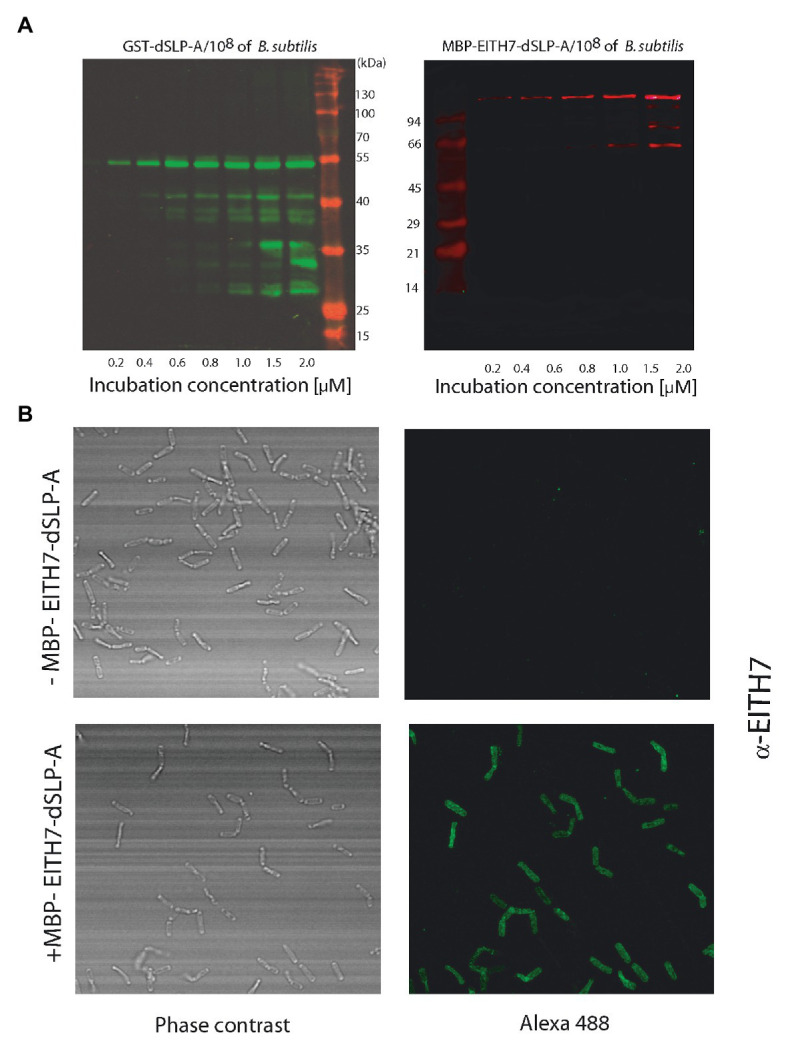
**(A)** Western blot analysis shows the increased binding of GST-dSLP-A (left) or MBP-EITH7-dSLP-A (right) to *Bacillus subtilis* surface that correlates with the increasing concentration of these proteins in the incubation solution. (Mk) Pre-stained molecular markers. **(B)** Confocal immunofluorescence microscopy of *B. subtilis* (upper panel) or *B. subtilis* decorated with MBP-EITH7-dSLP-A (lower panel) revealed with anti-EITH7 as the primary antibody.

Teichoic acid and wall teichoic acid which are present on the lactobacilli bacterial cell wall have been characterized as natural ligands for the dSLP-A domain (Fina Martin et al., 2019). Therefore, it is conceivable to expect that, in addition to *L. acidophilus*, dSLP-A-tagged proteins could bind also to any bacteria that display teichoic acids on its cell wall. Thus, dSLP-A-tagged proteins (GST-dSLP-A and M-EITH7-SLP-A) were also able to associate with *B. subtilis* var. natto (also a GRAS microorganism; Figure 4A). Association of the chimeric antigen M-EITH7-SLP-A to the surface of *B. subtilis* natto was also confirmed by confocal microscopy (Figure 4B).

### Mice Orally Immunized With *L. acidophilus* Decorated With M-EITH7-dSLP-A Elicited An Early Humoral and Mucosal Immune Response

**Figure 5:**
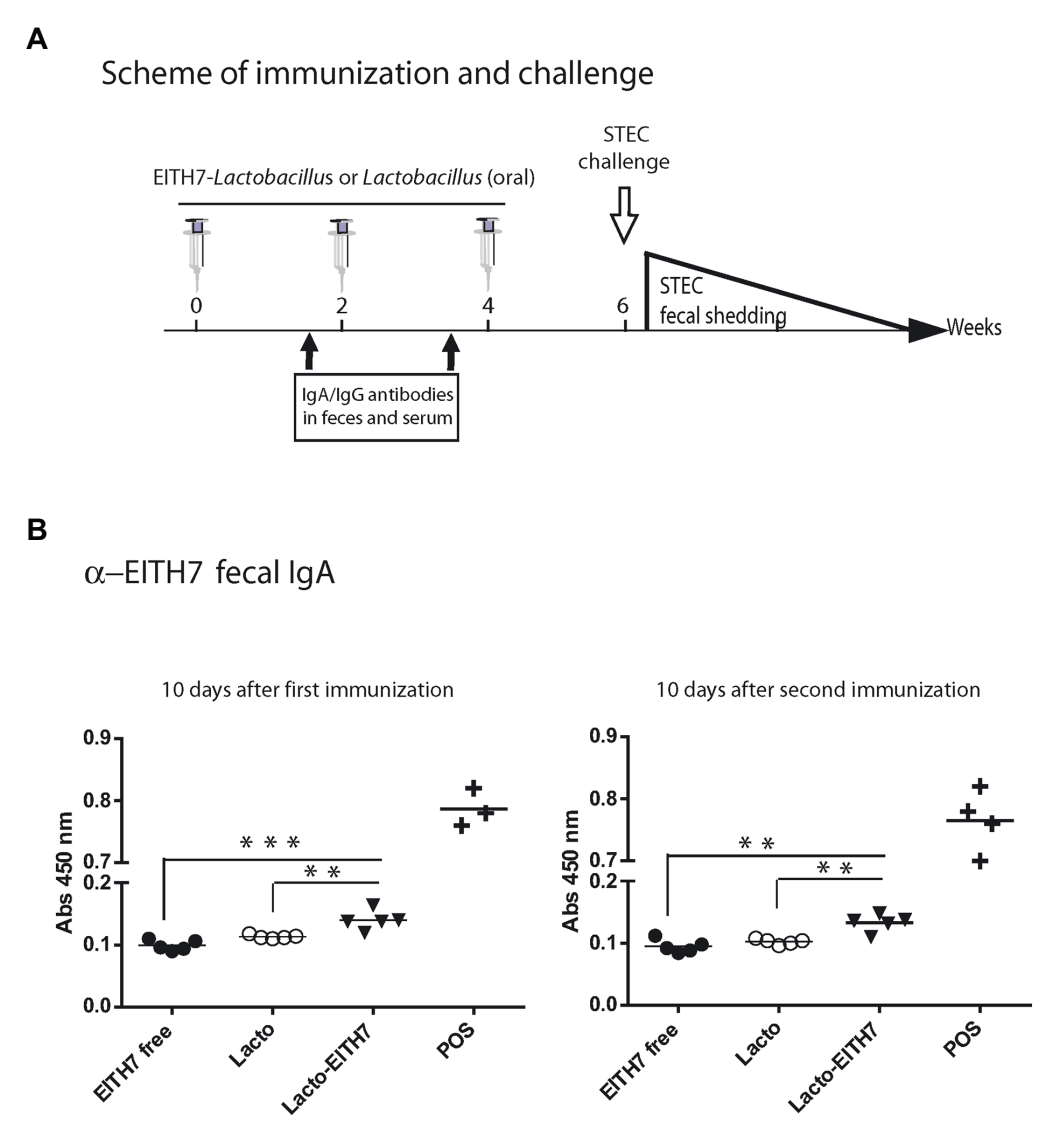
**(A)** Scheme of immunization and study of vaccine protection against a challenging infection in a mouse model. **(B)** BALB/c mice were orally immunized with free 20 μg of MBP-EITH7-dSLP-A, 4 × 10^8^ UFC of lactobacilli, or 4 × 10^8^ CFU of lactobacilli decorated with 20 μg of MBP-EITH7-dSLP-A in a scheme of three doses. Indirect ELISA for detection of mucosal IgA in feces (*n* = 6) was performed at 10 and 20 days post-immunization. A reference positive serum against EITH7 generated by i.p. immunization was used as a positive control (POS). One way ANOVA and Bonferroni’s Multiple Comparison *post hoc* Test (^*^p < 0.05), (^***^p < 0.01), and (^***^p < 0.001) was performed to analyze the statistical significance of results. Results shown are representative of three independent experiments.

**Figure 6:**
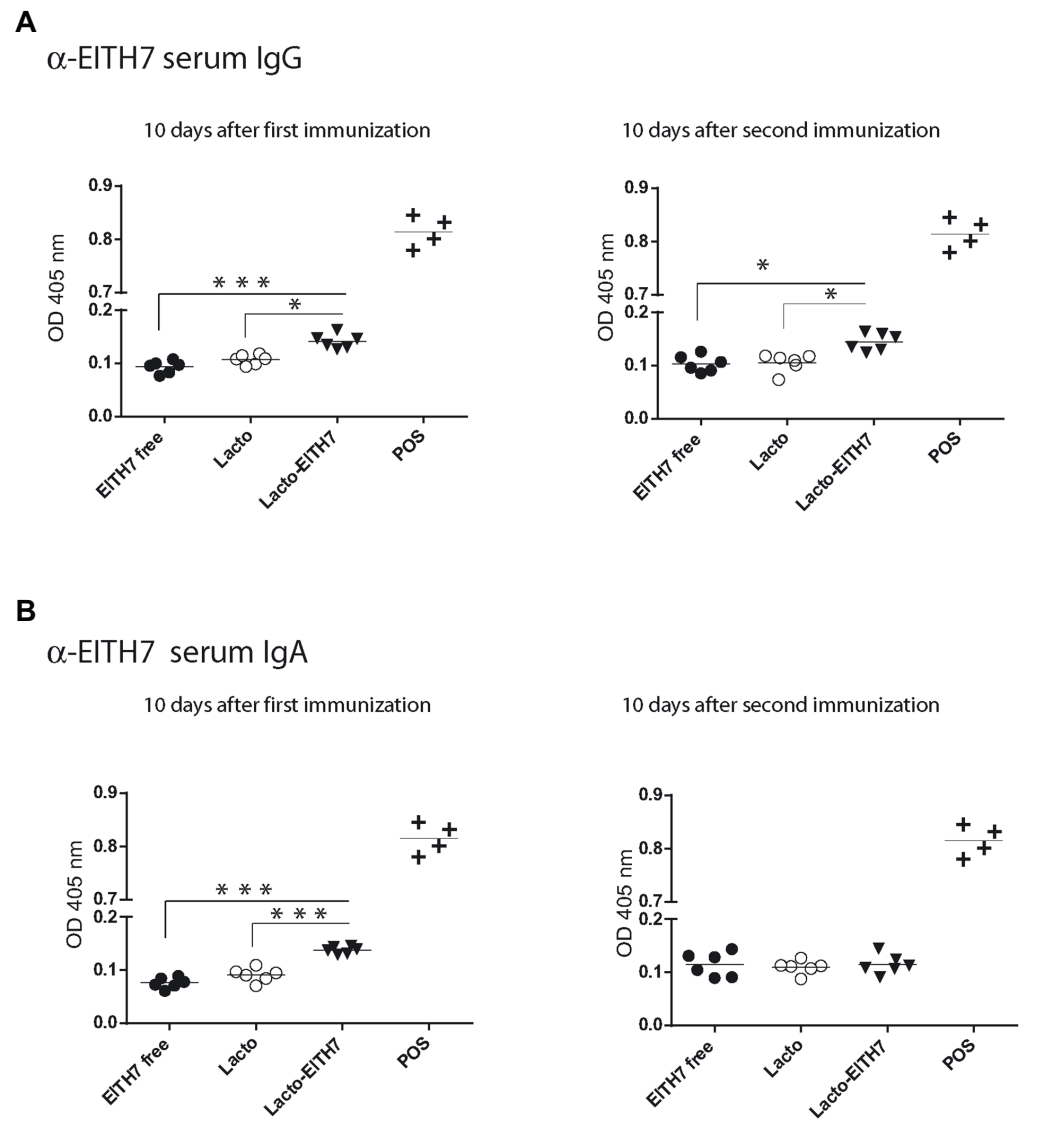
Indirect ELISA for the detection of serum anti-EITH7 specific IgG **(A)** and IgA **(B)** antibodies (*n* = 6). A reference positive serum against EITH7 generated by i.p. immunization, as described in the section Materials and Methods, was used as a POS. One way ANOVA and Bonferroni’s Multiple Comparison Test was used to analyze the statistical significance of results. Results shown are representative of three independent experiments. ^*^p < 0.05, ^**^p < 0.01, and ^***^p < 0.001.

Figure 5A shows a diagram of the immunization protocol used. BALB/c mice were immunized with three oral doses of different antigenic preparations (at 0, 2, and 4 weeks) and afterward (6 weeks post-immunization), all the immunized groups were experimentally challenged with 10^10^ CFU of *E. coli* O157:H7. Interestingly, after the first dose of M-EITH7-dSLP-A associated to *L. acidophilus* (10 days post-immunization), a significant increase in fecal EITH7-specific antibodies (IgA) was detected compared with the oral administration of the free M-EITH7-dSLP-A antigen or *L. acidophilus* alone used as a negative control. Similar results were also observed at 20 days after the first immunization (10 days after the second immunization; Figure 5B). In addition, the presence of EITH7-specific antibodies (IgG and IgA) in mouse sera was also determined by ELISA (Figure 6). A significant increase of anti-EITH7 titers in sera, IgG (Figure 6A), and IgA antibodies (Figure 6B) was detected 10 days post-immunization although at 20 days post-immunization only anti-EITH7 IgG was significantly increased. Of relevance, oral administration of *wild type L. acidophilus* that naturally carries a full-length SlpA (our negative control) or the administration of the free antigen M-EITH7-dSLP-A (Figure 6B, M-EITH7-dSLP-A_free_) elicited no significant increase in IgA titers in feces (Figure 5B) or in sera (Figure 6). Altogether, these results indicate that only oral administration of M-EITH7-dSLP-A carried by *L. acidophilus* was able to induce a significant mucosal and humoral immune response against the EITH7 antigen. These results suggest that the association of EITH7 to *L. acidophilus* allows the effective delivery of the antigen to the competent sites for an effective antigen presentation to induce a specific immune response.

### Mice Immunized With M-EITH7-dSLP-A- *L. acidophilus* Efficiently Control A STEC Challenge Infection

As shown in Figures 5, 6, induction of a mucosal and humoral immune response in mice required the administration of the antigen M-EITH7-dSLP-A bound to *L. acidophilus* while either the free M-EITH7-dSLP-A or *L. acidophilus* were not sufficient to elicit an EITH7-specific immune response. To determine if this antibody immune response correlated with an effective protection against an experimental infection with *E. coli* O157:H7, mice were immunized with different preparations and orally challenged with 10^10^ CFU of STEC.

**Figure 7:**
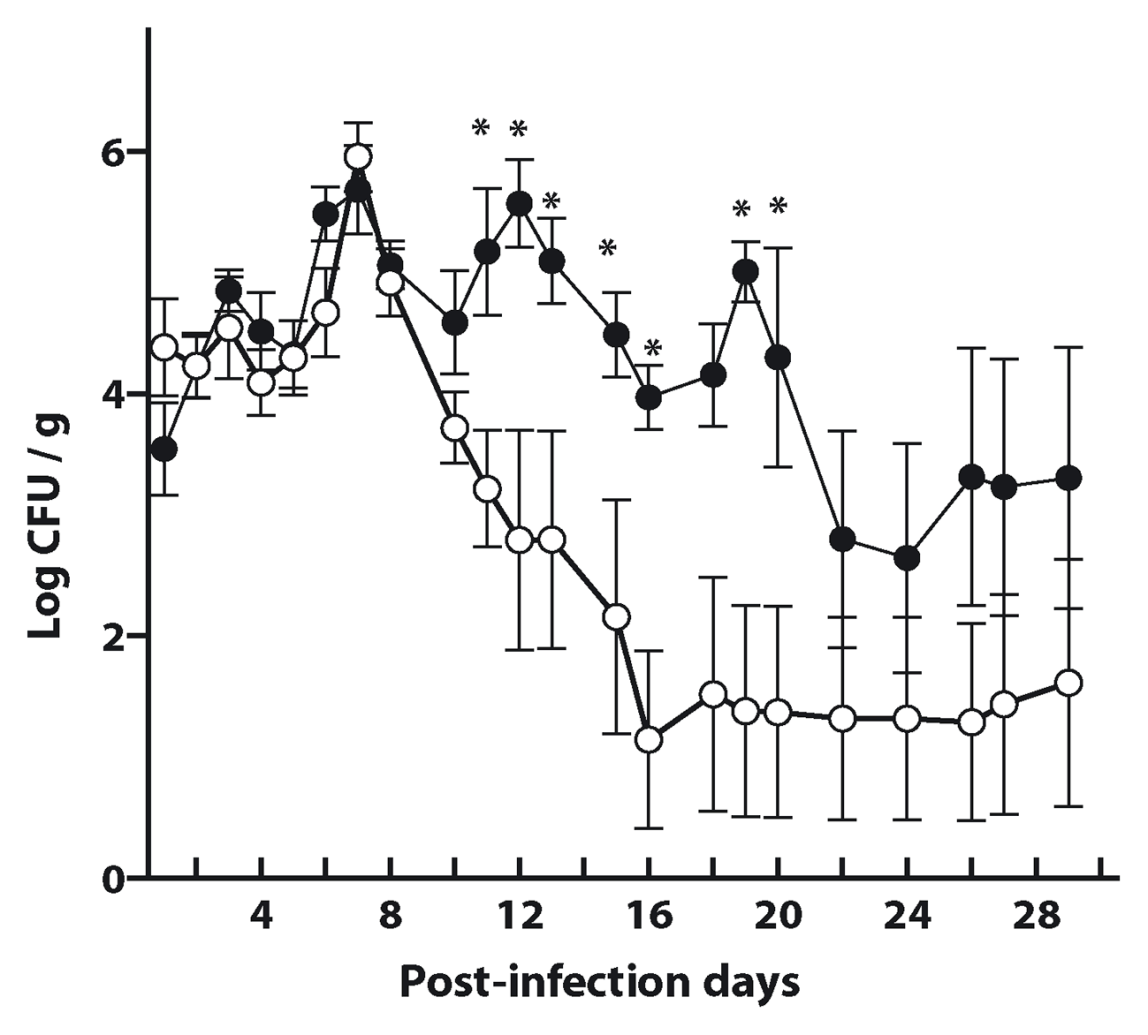
*L. acidophilus* and *L. acidophilus* decorated with EITH7-dSLP-A were administrated three times (0, 2, and 4 weeks) to mice. Ten days after the last immunization, mice were experimentally infected with 10^10^ CFU of *E. coli* O157:H7 and bacterial shedding in feces was monitored daily and the CFU per gram of feces was determined (*n* = 6). Results shown are representative of three independent experiments. Student’s *t*-test was used to analyze the statistical significance of results. ^*^p < 0.05.

Bacterial fecal shedding was determined to estimate the efficiency of protection elicited by the antigen preparation. As shown in Figure 7, mice immunized with either the antigen decorated *L. acidophilus*-(M-EITH7-dSLP-A; white dots) or with *L. acidophilus* (black dots) shed equivalent levels of STEC in mouse feces during the first-week post-challenge. Interestingly, after 11 days post-challenge, a significant reduction in fecal shedding was observed in the group that received *L. acidophilus*-(M-EITH7-dSLP-A) compared with the control group. Mice that received the carrier *L. acidophilus* alone kept shedding more than 10^4^ STEC/g in feces for almost 3 weeks post-challenge while the group that received antigen-decorated *L. acidophilus*-(M-EITH7-dSLP-A) shed less than 10^2^ STEC/g at the same time post-challenge. These results indicate that the EITH7 antigen delivered by *Lactobacillus* as a vaccine carrier was able to induce a protective immune response that correlates with the differential immune response observed.

## Discussion

It has been described that strains of *Lactobacillus* can stimulate the specific and the innate immune responses and, consequently, have been proposed as vaccine carriers (Pardo et al., 2016; Szatraj et al., 2017; Lecureux and Dean, 2018). Activation of the innate immune response is mediated by the PRRs expressed on immune cells that recognize conserved molecular structures known as MAMPs that activate the production of cytokines, chemokines, and other innate effectors. Host recognition of *Lactobacillus* is predominantly mediated by TLR2 receptor that recognizes teichoic and lipoteichoich acids which are present in the *Lactobacillus* cell wall leading to maturation of dendritic cells (Konstantinov et al., 2008) and the differentiation of monocytic precursors to Langerhans-like cells (Song et al., 2018). Also, it was shown that the *L. acidophilus* S-layer protein is able to interact with the DC-SIGN receptor (Dendritic Cell-Specific Intercellular adhesion molecule-3-Grabbing Non-integrin; CD209) a major dendritic cell receptor, interfering with the association of several human viruses such as HIV (Petrova et al., 2013) and JUNV (Martinez et al., 2012).

Here, we report the adaptation of *L. acidophilus* to deliver antigens to the gastrointestinal mucosa. Traditionally, *Lactobacillus* species have been engineered to express viral and bacterial antigens to elicit protective immune responses (Lecureux and Dean, 2018). This approach has a regulatory problem as it generates GMOs that are strictly regulated by several government offices (Directive 2001/18/EC of the European Parliament) if they will be released to the environment. In our system, antigens are passively attached to the surface of *L. acidophilus* with no alteration of the genotype and, therefore, with no generation of GMO microorganisms. For this reason, vaccine candidates based on this antigen decoration strategy are expected to have an easier, faster, and less-expensive regulatory approval process. Taking this into consideration, we studied if this strategy is suitable for the development of a vaccine candidate against the STEC. STEC infections can induce the development of the HUS, particularly in children below 5-year old that are the most susceptible population group (Fernandez-Brando et al., 2017). In addition to the severe consequences of HUS in children, it is also important to mention the economical burden that this disease represents for Public Health (e.g., STEC infection is a recurrent cause for kidney transplant in children and young people). Despite all these problems, there are no approved therapies or licensed vaccines against this bacterial infection for human use. The development of a safe oral vaccine could be of critical relevance for preventing HUS in children. Recently, two papers have proposed the adaptation of *Lactobacillus* for STEC vaccine development (Ferreira et al., 2011; Lin et al., 2017). In one of these reports, Lin et al. (2017) engineered *L. acidophilus* to express a chimeric STEC antigen composed by EspA and Tir (named E-T) with a signal peptide for the secretion to the bacterial supernatant. Interestingly, these authors have shown that E-T-vaccinated mice were protected against an experimental infection (10^10^ *E. coli* O157), similar to what we have shown here. Interestingly, although the fecal immune response (determined by ELISA signal) was similar to our results, immunization with E-T secreting-*Lactobacillus* induced an antibody immune response in sera than was relatively much stronger than the one we observed (Figure 6). It is interesting to speculate if these discrepancies between results could be reflecting differences in antigen dose since a recombinant strain of *Lactobacillus* has the capacity to express the heterologous antigens after it replicates. In our case, antigen-decorated bacteria are limited to a one-shot opportunity to deliver its cargo to the mucosal tissues, since the *de novo* expression of the recombinant antigen is not possible. This limitation could be sorted out either by optimizing the amount of antigen to be delivered increasing the number of decorated bacteria or by augmenting the number of doses. Since the amount of decorating antigen can be finely tuned and monitored it is possible to optimize the immunization protocol to induce a more effective immune response. Additionally, the system can be improved since it allows the co-decoration of *L. acidophilus* with proteins with different activities like immune modulators such as the TLR5-agonist of *Salmonella* flagellin, the lectin GAL8, or with the protease inhibitor *B. abortus* Omp19. Interestingly, all these molecules can modulate the induction of the immune response by altering the way that antigens are presented to the immune system, enhancing its half-life, or providing a particular environment of cytokines for a more efficient antigen presentation. As shown here, the fusion of the dSLP-A domains to all protein tested allows the adhesion of the resulting chimera protein to the surface of *L. acidophilus*. As shown in Figure 4, in addition to *L. acidophilus*, dSLP-A-tagged proteins can also bind to different bacteria that display teichoic acids on its cell wall such as *B. subtilis*. This could be particularly useful to optimize a prime/boost immunization strategy by simply switching the bacterial carrier when a second dose is required. BLAST sequence analysis showed that *L. acidophilus* dSLP-A domain displays more than 75% amino acid identity to SlpA sequences present in *L. helveticus*, *L. crispatus*, *L. amyolovorus*, and *L. gallinarum* (Supplementary Table S1). Since *L. helveticus* and *L. crispatus* are vaginal mucosa resident bacteria (Nunn and Forney, 2016), it could be interesting to explore if they can be adapted as vaccine carriers for the induction of protective immune responses at genital mucosa against sexually transmitted diseases like HBV, HPV, or HIV.

In this report, and as a proof of concept, we demonstrated that *L. acidophilus* could be adapted as a vaccine carrier by coating the bacteria with antigens from a microbial pathogen mimicking its surface, to induce a protective immune response for vaccination purposes.

## Conclusion

Here, we have explored the adaptation of a GRAS bacterium such as *L. acidophilus* as an antigen delivery system. Decoration of the *L. acidophilus* with heterologous proteins might allow the development of a universal platform for intestinal delivery of proteins or enzymes with therapeutic value. Of interest, all these biotechnological applications will be achieved without the generation of genetically modified bacteria or the introduction of antibiotic resistances.

## Data Availability Statement

All datasets generated for this study are included in the article/Supplementary Material.

## Ethics Statement

The animal study was reviewed and approved by Experimental procedure of this study (permit number 15/2018) was approved by the Committee on the Ethics of Animal Experiments of the Universidad Nacional de San Martín (UNSAM), under the recommendations for animal experimentation (Helsinki Declaration and its amendments, Amsterdam Protocol of welfare and animal protection and National Institutes of Health, USA NIH, guidelines: Guide for the Care and Use of Laboratory Animals).

## Author Contributions

SR, MR, and GB conceived and design the experiments. PU, CT, MP, and JM performed experiments. PU, MR, and GB analyzed data. SR and MP contribute with reagent, material. MR and GB wrote the paper. All authors contributed to the article and approved the submitted version.

### Conflict of Interest

The authors declare that the research was conducted in the absence of any commercial or financial relationships that could be construed as a potential conflict of interest.
