# Supplementary figures and images for: Development of an Antigen Delivery Platform Using Lactobacillus acidophilus Decorated With Heterologous Proteins: A Sheep in Wolf’s Clothing Story
Source: Front Microbiol. 2020 Oct 27;11:509380. doi: 10.3389/fmicb.2020.509380 (PMC7652789; doi:10.3389/fmicb.2020.509380)

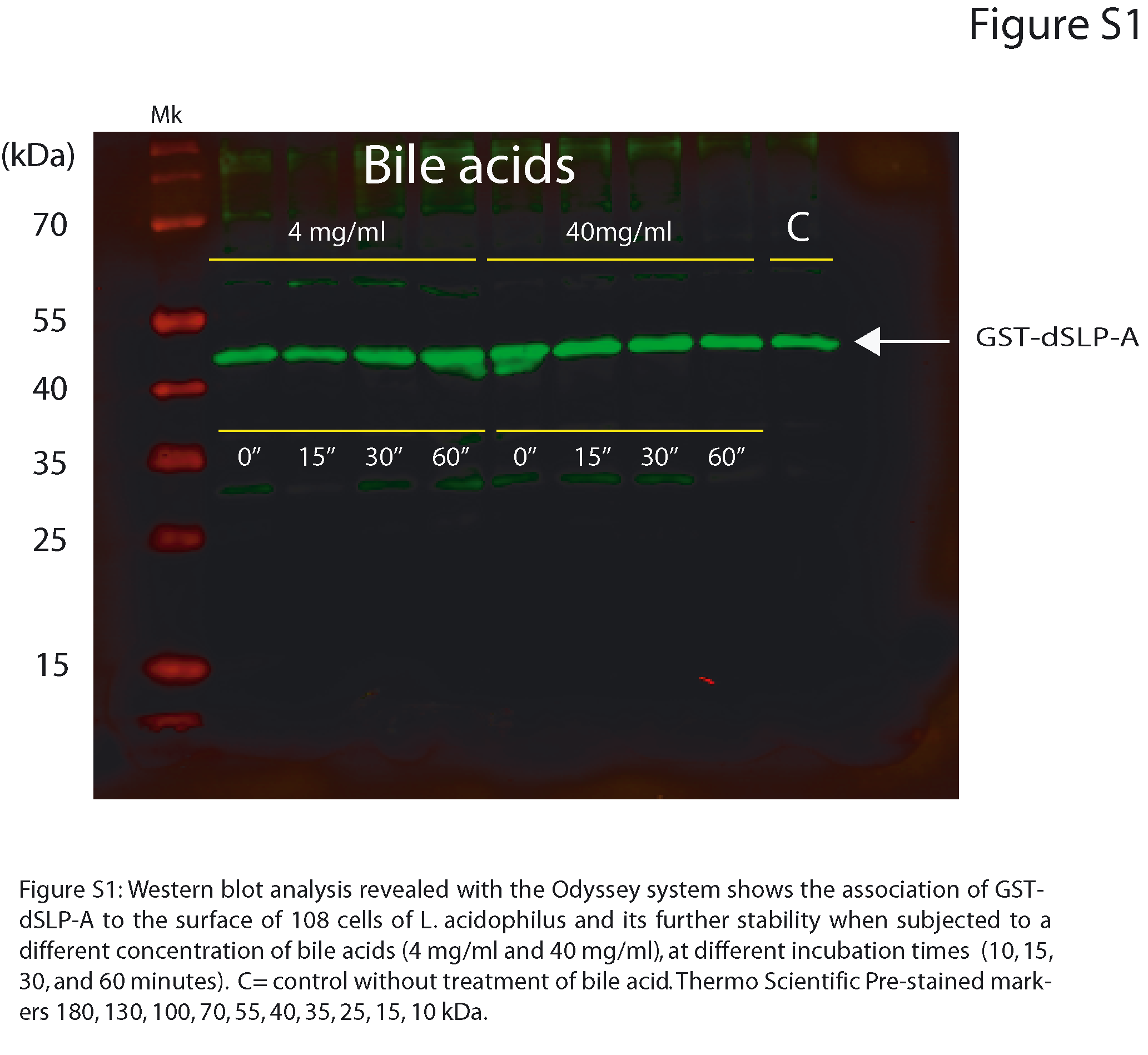

Supplement: Supplementary file 1 [file Image_1.tif]
