# Supplementary material for: Development of an Antigen Delivery Platform Using Lactobacillus acidophilus Decorated With Heterologous Proteins: A Sheep in Wolf’s Clothing Story
Source: Front Microbiol. 2020 Oct 27;11:509380. doi: 10.3389/fmicb.2020.509380 (PMC7652789; doi:10.3389/fmicb.2020.509380)
Supplement: Supplementary file 2 [file Data_Sheet_1.pdf]

\TABLE S1



**BLAST ®** » **blastp suite** » **results for RID-RCMHK1VT015**

Your results are filtered to match records with percent identity between 75 and 100.

| | |
|---|---|
| **Job Title** | Protein Sequence ... |
| **RID** | RCMHK1VT015 Search expires on 09-10 21:22 pm |
| **Program** | BLASTP |
| **Database** | nr |
| **Query ID** | lcl\|Query_205445 |
| **Description** | None ... |
| **Molecule type** | amino acid |
| **Query Length** | 159 |

## Descriptions

| Description | E value | Per. Ident |
|---|---|---|
| S-layer protein [Lactobacillus acidophilus] | 2e-105 | 100.00% |
| S-layer protein [Lactobacillus acidophilus] | 2e-105 | 100.00% |
| S-layer protein [Lactobacillus acidophilus] | 3e-105 | 100.00% |
| S-layer protein [Lactobacillus acidophilus] | 3e-105 | 100.00% |
| S-layer protein [Lactobacillus acidophilus] | 3e-101 | 100.00% |
| S-layer protein [Lactobacillus acidophilus] | 8e-89 | 100.00% |
| S-layer protein [Lactobacillus acidophilus] | 3e-74 | 100.00% |
| S-layer protein [Lactobacillus acidophilus] | 1e-104 | 99.37% |
| S-layer protein [Lactobacillus acidophilus] | 2e-104 | 99.37% |
| S-layer protein [Lactobacillus acidophilus] | 9e-80 | 99.19% |
| surface layer protein [Lactobacillus acidophilus] | 3e-89 | 89.87% |
| S-layer protein [Lactobacillus acidophilus] | 1e-88 | 89.31% |
| S-layer protein [Lactobacillus acidophilus] | 2e-88 | 89.31% |
| S-layer protein [Lactobacillus acidophilus] | 5e-85 | 89.03% |
| S-layer protein [Lactobacillus acidophilus] | 8e-88 | 88.68% |
| S-layer protein [Lactobacillus acidophilus] | 8e-88 | 88.68% |
| SB-protein [Lactobacillus acidophilus] | 3e-86 | 87.42% |
| surface layer protein [Lactobacillus helveticus] | 1e-77 | 79.38% |
| S-layer protein [Lactobacillus helveticus] | 1e-76 | 78.75% |
| RecName: Full=S-layer protein; AltName: Full=Surface layer protein; Flags: Precursor [Lactobacillus helveticus] | 1e-76 | 78.75% |
| S-layer protein [Lactobacillus helveticus] | 1e-76 | 78.75% |
| S-layer protein [Lactobacillus helveticus] | 1e-76 | 78.75% |
| surface layer protein [Lactobacillus helveticus] | 1e-76 | 78.75% |
| surface layer protein [Lactobacillus helveticus] | 1e-76 | 78.75% |
| surface layer protein [Lactobacillus helveticus] | 1e-76 | 78.75% |
| S-layer protein [Lactobacillus helveticus] | 1e-76 | 78.75% |
| surface layer protein [Lactobacillus helveticus DSM 20075 = CGMCC 1.1877] | 1e-76 | 78.75% |
| surface layer protein [Lactobacillus helveticus] | 2e-76 | 78.75% |
| S-layer protein [Lactobacillus helveticus] | 2e-76 | 78.75% |
| S-layer protein [Lactobacillus helveticus] | 2e-76 | 78.75% |
| S-layer protein [Lactobacillus helveticus] | 2e-76 | 78.75% |
| surface layer protein [Lactobacillus helveticus] | 2e-76 | 78.75% |
| S-layer protein [Lactobacillus helveticus] | 4e-76 | 78.75% |
| S-layer protein [Lactobacillus helveticus] | 4e-76 | 78.75% |
| S-layer protein [Lactobacillus helveticus] | 4e-76 | 78.75% |
| surface layer protein [Lactobacillus helveticus] | 2e-75 | 78.62% |
| surface layer protein [Lactobacillus helveticus] | 2e-76 | 78.12% |
| surface layer protein [Lactobacillus helveticus] | 7e-76 | 78.12% |
| S-layer protein [Lactobacillus amylovorus] | 6e-76 | 77.99% |
| unnamed protein product | 8e-75 | 77.99% |

| Description | E value | Per. Ident |
|---|---|---|
| S-layer protein [Lactobacillus helveticus] | 2e-74 | 77.99% |
| S-layer protein [Lactobacillus helveticus] | 1e-73 | 77.36% |
| Surface layer protein [Lactobacillus helveticus H10] | 3e-73 | 77.36% |
| S-layer protein [Lactobacillus kefiranofaciens] | 5e-76 | 77.22% |
| S-layer protein [Lactobacillus gallinarum] | 2e-73 | 77.07% |
| S-layer protein [Lactobacillus helveticus] | 5e-73 | 76.73% |
| S-layer protein [Lactobacillus gallinarum] | 9e-73 | 76.73% |
| S-layer protein [Lactobacillus amylovorus] | 1e-67 | 76.43% |
| surface layer protein [Lactobacillus helveticus] | 9e-71 | 76.13% |
| S-layer protein [Lactobacillus amylovorus] | 5e-73 | 75.80% |
| S-layer protein [Lactobacillus amylovorus] | 2e-71 | 75.80% |
| S-layer protein [Lactobacillus helveticus] | 4e-71 | 75.80% |
| hypothetical protein [Lactobacillus helveticus] | 5e-69 | 75.48% |
| S-layer protein [Lactobacillus helveticus] | 5e-69 | 75.48% |
| S-layer protein [Lactobacillus helveticus] | 5e-69 | 75.48% |
| surface layer protein [Lactobacillus helveticus] | 6e-69 | 75.48% |
| S-layer protein [Lactobacillus helveticus] | 6e-69 | 75.48% |
| hypothetical protein [Lactobacillus helveticus] | 6e-69 | 75.48% |
| Surface layer protein [Lactobacillus helveticus DPC 4571] | 1e-68 | 75.48% |
| S-layer protein [Lactobacillus crispatus] | 3e-72 | 75.32% |
| hypothetical protein [Lactobacillus amylovorus] | 3e-72 | 75.32% |
| S-layer protein [Lactobacillus crispatus] | 1e-71 | 75.32% |
| S-layer protein [Lactobacillus amylovorus] | 3e-71 | 75.16% |

## Graphic Summary

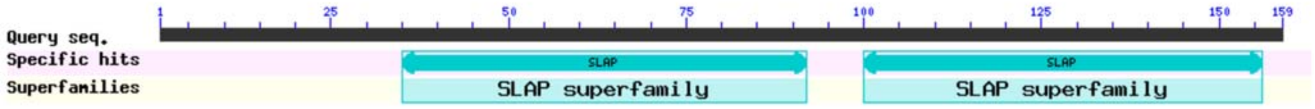

Distribution of the top 63 Blast Hits on 63 subject sequences

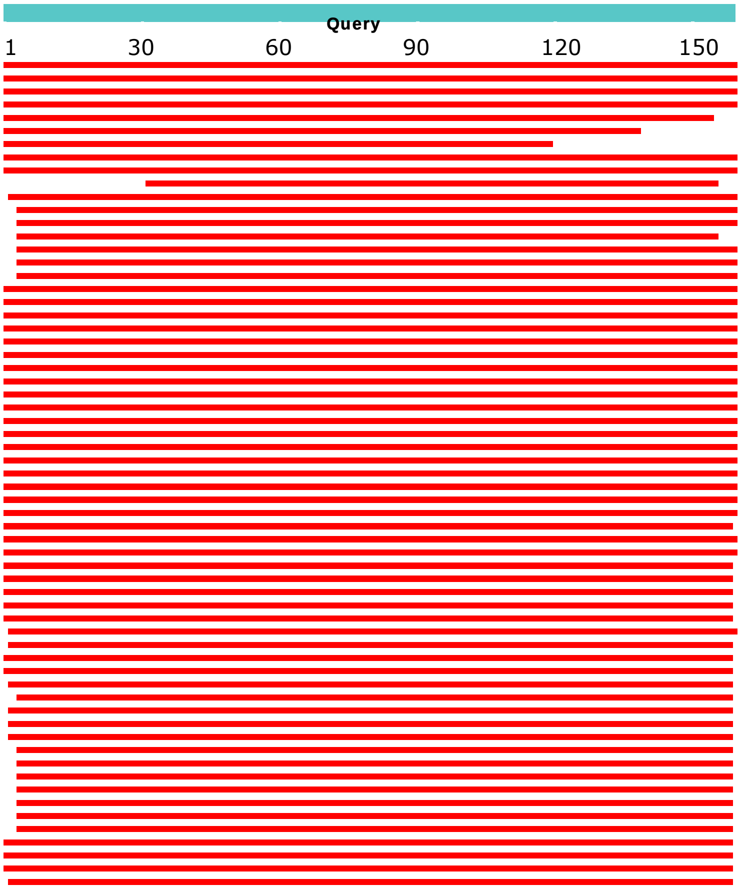


## Alignments

Alignment view Pairwise CDS feature

S-layer protein [Lactobacillus acidophilus]

Sequence ID: **WP_125977721.1** Length: 446 Number of Matches: 1

Range 1: 288 to 446

| Score | Expect | Method | Identities | Positives | Gaps | Frame |
|---|---|---|---|---|---|---|
| 318 bits(814) | 2e-105() | Compositional matrix adjust. | 159/159(100%) | 159/159(100%) | 0/159(0%) | |

```
Query  1    NVKATSNTNGKSATLPVVVTVPNVAEPTVASVSKRIMHNAYYYDKDAKRVGTDSVKRYNS  60
            NVKATSNTNGKSATLPVVVTVPNVAEPTVASVSKRIMHNAYYYDKDAKRVGTDSVKRYNS
Sbjct  288  NVKATSNTNGKSATLPVVVTVPNVAEPTVASVSKRIMHNAYYYDKDAKRVGTDSVKRYNS  347

Query  61   VSVLPNTTTINGKTYYQVVENGKAVDKYINAANIDGTKRTLKHNAYVYASSKKRANKVVL  120
            VSVLPNTTTINGKTYYQVVENGKAVDKYINAANIDGTKRTLKHNAYVYASSKKRANKVVL
Sbjct  348  VSVLPNTTTINGKTYYQVVENGKAVDKYINAANIDGTKRTLKHNAYVYASSKKRANKVVL  407

Query  121  KKGEVVTTYGASYTFKNGQKYYKIGDNTDKTYVKVANFR  159
            KKGEVVTTYGASYTFKNGQKYYKIGDNTDKTYVKVANFR
Sbjct  408  KKGEVVTTYGASYTFKNGQKYYKIGDNTDKTYVKVANFR  446
```

S-layer protein [Lactobacillus acidophilus]

Sequence ID: **WP_075917333.1** Length: 447 Number of Matches: 1

Range 1: 289 to 447

| Score | Expect | Method | Identities | Positives | Gaps | Frame |
|---|---|---|---|---|---|---|
| 317 bits(813) | 2e-105() | Compositional matrix adjust. | 159/159(100%) | 159/159(100%) | 0/159(0%) | |

```
Query  1    NVKATSNTNGKSATLPVVVTVPNVAEPTVASVSKRIMHNAYYYDKDAKRVGTDSVKRYNS  60
            NVKATSNTNGKSATLPVVVTVPNVAEPTVASVSKRIMHNAYYYDKDAKRVGTDSVKRYNS
Sbjct  289  NVKATSNTNGKSATLPVVVTVPNVAEPTVASVSKRIMHNAYYYDKDAKRVGTDSVKRYNS  348

Query  61   VSVLPNTTTINGKTYYQVVENGKAVDKYINAANIDGTKRTLKHNAYVYASSKKRANKVVL  120
            VSVLPNTTTINGKTYYQVVENGKAVDKYINAANIDGTKRTLKHNAYVYASSKKRANKVVL
Sbjct  349  VSVLPNTTTINGKTYYQVVENGKAVDKYINAANIDGTKRTLKHNAYVYASSKKRANKVVL  408

Query  121  KKGEVVTTYGASYTFKNGQKYYKIGDNTDKTYVKVANFR  159
            KKGEVVTTYGASYTFKNGQKYYKIGDNTDKTYVKVANFR
Sbjct  409  KKGEVVTTYGASYTFKNGQKYYKIGDNTDKTYVKVANFR  447
```

S-layer protein [Lactobacillus acidophilus]

Sequence ID: **WP_089541165.1** Length: 444 Number of Matches: 1
Range 1: 286 to 444

| Score | Expect | Method | Identities | Positives | Gaps | Frame |
|---|---|---|---|---|---|---|
| 317 bits(812) | 3e-105() | Compositional matrix adjust. | 159/159(100%) | 159/159(100%) | 0/159(0%) | |

```
Query  1    NVKATSNTNGKSATLPVVVTVPNVAEPTVASVSKRIMHNAYYYDKDAKRVGTDSVKRYNS  60
            NVKATSNTNGKSATLPVVVTVPNVAEPTVASVSKRIMHNAYYYDKDAKRVGTDSVKRYNS
Sbjct  286  NVKATSNTNGKSATLPVVVTVPNVAEPTVASVSKRIMHNAYYYDKDAKRVGTDSVKRYNS  345

Query  61   VSVLPNTTTINGKTYYQVVENGKAVDKYINAANIDGTKRTLKHNAYVYASSKKRANKVVL  120
            VSVLPNTTTINGKTYYQVVENGKAVDKYINAANIDGTKRTLKHNAYVYASSKKRANKVVL
Sbjct  346  VSVLPNTTTINGKTYYQVVENGKAVDKYINAANIDGTKRTLKHNAYVYASSKKRANKVVL  405

Query  121  KKGEVVTTYGASYTFKNGQKYYKIGDNTDKTYVKVANFR  159
            KKGEVVTTYGASYTFKNGQKYYKIGDNTDKTYVKVANFR
Sbjct  406  KKGEVVTTYGASYTFKNGQKYYKIGDNTDKTYVKVANFR  444
```

S-layer protein [Lactobacillus acidophilus]

Sequence ID: **WP_011254065.1** Length: 444 Number of Matches: 1
Range 1: 286 to 444

| Score | Expect | Method | Identities | Positives | Gaps | Frame |
|---|---|---|---|---|---|---|
| 317 bits(812) | 3e-105() | Compositional matrix adjust. | 159/159(100%) | 159/159(100%) | 0/159(0%) | |

```
Query  1    NVKATSNTNGKSATLPVVVTVPNVAEPTVASVSKRIMHNAYYYDKDAKRVGTDSVKRYNS  60
            NVKATSNTNGKSATLPVVVTVPNVAEPTVASVSKRIMHNAYYYDKDAKRVGTDSVKRYNS
Sbjct  286  NVKATSNTNGKSATLPVVVTVPNVAEPTVASVSKRIMHNAYYYDKDAKRVGTDSVKRYNS  345

Query  61   VSVLPNTTTINGKTYYQVVENGKAVDKYINAANIDGTKRTLKHNAYVYASSKKRANKVVL  120
            VSVLPNTTTINGKTYYQVVENGKAVDKYINAANIDGTKRTLKHNAYVYASSKKRANKVVL
Sbjct  346  VSVLPNTTTINGKTYYQVVENGKAVDKYINAANIDGTKRTLKHNAYVYASSKKRANKVVL  405

Query  121  KKGEVVTTYGASYTFKNGQKYYKIGDNTDKTYVKVANFR  159
            KKGEVVTTYGASYTFKNGQKYYKIGDNTDKTYVKVANFR
Sbjct  406  KKGEVVTTYGASYTFKNGQKYYKIGDNTDKTYVKVANFR  444
```

S-layer protein, partial [Lactobacillus acidophilus]

Sequence ID: **WP_063720662.1** Length: 439 Number of Matches: 1
Range 1: 286 to 439

| Score | Expect | Method | Identities | Positives | Gaps | Frame |
|---|---|---|---|---|---|---|
| 306 bits(785) | 3e-101() | Compositional matrix adjust. | 154/154(100%) | 154/154(100%) | 0/154(0%) | |

```
Query  1    NVKATSNTNGKSATLPVVVTVPNVAEPTVASVSKRIMHNAYYYDKDAKRVGTDSVKRYNS  60
            NVKATSNTNGKSATLPVVVTVPNVAEPTVASVSKRIMHNAYYYDKDAKRVGTDSVKRYNS
Sbjct  286  NVKATSNTNGKSATLPVVVTVPNVAEPTVASVSKRIMHNAYYYDKDAKRVGTDSVKRYNS  345

Query  61   VSVLPNTTTINGKTYYQVVENGKAVDKYINAANIDGTKRTLKHNAYVYASSKKRANKVVL  120
            VSVLPNTTTINGKTYYQVVENGKAVDKYINAANIDGTKRTLKHNAYVYASSKKRANKVVL
Sbjct  346  VSVLPNTTTINGKTYYQVVENGKAVDKYINAANIDGTKRTLKHNAYVYASSKKRANKVVL  405

Query  121  KKGEVVTTYGASYTFKNGQKYYKIGDNTDKTYVK  154
            KKGEVVTTYGASYTFKNGQKYYKIGDNTDKTYVK
Sbjct  406  KKGEVVTTYGASYTFKNGQKYYKIGDNTDKTYVK  439
```

S-layer protein, partial [Lactobacillus acidophilus]

Sequence ID: **WP_042710617.1** Length: 395 Number of Matches: 1
Range 1: 258 to 395

| Score | Expect | Method | Identities | Positives | Gaps | Frame |
|---|---|---|---|---|---|---|
| 274 bits(700) | 8e-89() | Compositional matrix adjust. | 138/138(100%) | 138/138(100%) | 0/138(0%) | |

```
Query  1    NVKATSNTNGKSATLPVVVTVPNVAEPTVASVSKRIMHNAYYYDKDAKRVGTDSVKRYNS  60
            NVKATSNTNGKSATLPVVVTVPNVAEPTVASVSKRIMHNAYYYDKDAKRVGTDSVKRYNS
Sbjct  258  NVKATSNTNGKSATLPVVVTVPNVAEPTVASVSKRIMHNAYYYDKDAKRVGTDSVKRYNS  317

Query  61   VSVLPNTTTINGKTYYQVVENGKAVDKYINAANIDGTKRTLKHNAYVYASSKKRANKVVL  120
            VSVLPNTTTINGKTYYQVVENGKAVDKYINAANIDGTKRTLKHNAYVYASSKKRANKVVL
Sbjct  318  VSVLPNTTTINGKTYYQVVENGKAVDKYINAANIDGTKRTLKHNAYVYASSKKRANKVVL  377

Query  121  KKGEVVTTYGASYTFKNG  138
            KKGEVVTTYGASYTFKNG
Sbjct  378  KKGEVVTTYGASYTFKNG  395
```

S-layer protein, partial [Lactobacillus acidophilus]

Sequence ID: **WP_029779823.1** Length: 381 Number of Matches: 1

Range 1: 263 to 381

| Score | Expect | Method | Identities | Positives | Gaps | Frame |
|---|---|---|---|---|---|---|
| 236 bits(602) | 3e-74() | Compositional matrix adjust. | 119/119(100%) | 119/119(100%) | 0/119(0%) | |

```
Query  1    NVKATSNTNGKSATLPVVVTVPNVAEPTVASVSKRIMHNAYYYDKDAKRVGTDSVKRYNS  60
            NVKATSNTNGKSATLPVVVTVPNVAEPTVASVSKRIMHNAYYYDKDAKRVGTDSVKRYNS
Sbjct  263  NVKATSNTNGKSATLPVVVTVPNVAEPTVASVSKRIMHNAYYYDKDAKRVGTDSVKRYNS  322

Query  61   VSVLPNTTTINGKTYYQVVENGKAVDKYINAANIDGTKRTLKHNAYVYASSKKRANKVV  119
            VSVLPNTTTINGKTYYQVVENGKAVDKYINAANIDGTKRTLKHNAYVYASSKKRANKVV
Sbjct  323  VSVLPNTTTINGKTYYQVVENGKAVDKYINAANIDGTKRTLKHNAYVYASSKKRANKVV  381
```

S-layer protein [Lactobacillus acidophilus]

Sequence ID: **WP_021721641.1** Length: 447 Number of Matches: 1

Range 1: 289 to 447

| Score | Expect | Method | Identities | Positives | Gaps | Frame |
|---|---|---|---|---|---|---|
| 316 bits(809) | 1e-104() | Compositional matrix adjust. | 158/159(99%) | 158/159(99%) | 0/159(0%) | |

```
Query  1    NVKATSNTNGKSATLPVVVTVPNVAEPTVASVSKRIMHNAYYYDKDAKRVGTDSVKRYNS  60
            NVKATSNTNGKSATLPVVVTVPNVAEPTVASVSKRIMHNAYYYDKDAKRVGTDSVKRYNS
Sbjct  289  NVKATSNTNGKSATLPVVVTVPNVAEPTVASVSKRIMHNAYYYDKDAKRVGTDSVKRYNS  348

Query  61   VSVLPNTTTINGKTYYQVVENGKAVDKYINAANIDGTKRTLKHNAYVYASSKKRANKVVL  120
            VSVLPNTTTINGK YYQVVENGKAVDKYINAANIDGTKRTLKHNAYVYASSKKRANKVVL
Sbjct  349  VSVLPNTTTINGKAYYQVVENGKAVDKYINAANIDGTKRTLKHNAYVYASSKKRANKVVL  408

Query  121  KKGEVVTTYGASYTFKNGQKYYKIGDNTDKTYVKVANFR  159
            KKGEVVTTYGASYTFKNGQKYYKIGDNTDKTYVKVANFR
Sbjct  409  KKGEVVTTYGASYTFKNGQKYYKIGDNTDKTYVKVANFR  447
```

S-layer protein [Lactobacillus acidophilus]

Sequence ID: **WP_021721262.1** Length: 444 Number of Matches: 1

Range 1: 286 to 444

| Score | Expect | Method | Identities | Positives | Gaps | Frame |
|---|---|---|---|---|---|---|
| 315 bits(808) | 2e-104() | Compositional matrix adjust. | 158/159(99%) | 158/159(99%) | 0/159(0%) | |

```
Query  1    NVKATSNTNGKSATLPVVVTVPNVAEPTVASVSKRIMHNAYYYDKDAKRVGTDSVKRYNS  60
            NVKATSNTNGKSATLPVVVTVPNVAEPTVASVSKRIMHNAYYYDKDAKRVGTDSVKRYNS
Sbjct  286  NVKATSNTNGKSATLPVVVTVPNVAEPTVASVSKRIMHNAYYYDKDAKRVGTDSVKRYNS  345

Query  61   VSVLPNTTTINGKTYYQVVENGKAVDKYINAANIDGTKRTLKHNAYVYASSKKRANKVVL  120
            VSVLPNTTTINGK YYQVVENGKAVDKYINAANIDGTKRTLKHNAYVYASSKKRANKVVL
Sbjct  346  VSVLPNTTTINGKAYYQVVENGKAVDKYINAANIDGTKRTLKHNAYVYASSKKRANKVVL  405

Query  121  KKGEVVTTYGASYTFKNGQKYYKIGDNTDKTYVKVANFR  159
            KKGEVVTTYGASYTFKNGQKYYKIGDNTDKTYVKVANFR
Sbjct  406  KKGEVVTTYGASYTFKNGQKYYKIGDNTDKTYVKVANFR  444
```

S-layer protein, partial [Lactobacillus acidophilus]

Sequence ID: **PTV27709.1** Length: 124 Number of Matches: 1

Range 1: 1 to 124

| Score | Expect | Method | Identities | Positives | Gaps | Frame |
|---|---|---|---|---|---|---|
| 241 bits(615) | 9e-80() | Compositional matrix adjust. | 123/124(99%) | 123/124(99%) | 0/124(0%) | |

```
Query  32   VSKRIMHNAYYYDKDAKRVGTDSVKRYNSVSVLPNTTTINGKTYYQVVENGKAVDKYINA  91
            VSKRIMHNAYYYDKDAKRVGTDSVKRYNSVSVLPNTTTINGK YYQVVENGKAVDKYINA
Sbjct  1    VSKRIMHNAYYYDKDAKRVGTDSVKRYNSVSVLPNTTTINGKAYYQVVENGKAVDKYINA  60

Query  92   ANIDGTKRTLKHNAYVYASSKKRANKVVLKKGEVVTTYGASYTFKNGQKYYKIGDNTDKT  151
            ANIDGTKRTLKHNAYVYASSKKRANKVVLKKGEVVTTYGASYTFKNGQKYYKIGDNTDKT
Sbjct  61   ANIDGTKRTLKHNAYVYASSKKRANKVVLKKGEVVTTYGASYTFKNGQKYYKIGDNTDKT  120

Query  152  YVKV  155
            YVKV
Sbjct  121  YVKV  124
```

surface layer protein [Lactobacillus acidophilus]

Sequence ID: **AEW12794.1** Length: 467 Number of Matches: 1

Range 1: 310 to 467

| Score | Expect | Method | Identities | Positives | Gaps | Frame |
|---|---|---|---|---|---|---|
| 277 bits(709) | 3e-89() | Compositional matrix adjust. | 142/158(90%) | 145/158(91%) | 0/158(0%) | |

```
Query  2    VKATSNTNGKSATLPVVVTVPNVAEPTVASVSKRIMHNAYYYDKDAKRVGTDSVKRYNSV  61
            + A S  NGK ATLPV VTVPN    TV SVSKRIMHNAY+YDKDAKRVGTDSVKRY SV
Sbjct  310  LTAKSEVNGKVATLPVTVTVPNGKVTTVDSVSKRIMHNAYFYDKDAKRVGTDSVKRYASV  369
```

```
Query  62   SVLPNTTTINGKTYYQVVENGKAVDKYINAANIDGTKRTLKHNAYVYASSKKRANKVVLK  121
            SVLPNTTTINGK YYQVVENGKAVDKYINAANIDGTKRTLKHNAYVYASSKKRANKVVLK
Sbjct  370  SVLPNTTTINGKAYYQVVENGKAVDKYINAANIDGTKRTLKHNAYVYASSKKRANKVVLK  429

Query  122  KGEVVTTYGASYTFKNGQKYYKIGDNTDKTYVKVANFR  159
            KGEVVTTYGASYTFKNGQKYYKIG+NTDKTYVKVANFR
Sbjct  430  KGEVVTTYGASYTFKNGQKYYKIGNNTDKTYVKVANFR  467
```

S-layer protein [Lactobacillus acidophilus]

Sequence ID: **WP_125977729.1** Length: 457 Number of Matches: 1

Range 1: 299 to 457

| Score | Expect | Method | Identities | Positives | Gaps | Frame |
|---|---|---|---|---|---|---|
| 275 bits(704) | 1e-88() | Compositional matrix adjust. | 142/159(89%) | 145/159(91%) | 3/159(1%) | |

```
Query  4    ATSNTNGKSATLPVVVTVPN---VAEPTVASVSKRIMHNAYYYDKDAKRVGTDSVKRYNS  60
            A S  N ++A LPV V+V N   V   TV SVSKRIMHNAYYYDKDAKRVGTDSVKRYNS
Sbjct  299  AESTQNNETAQLPVTVSVTNGKEVTPSTVDSVSKRIMHNAYYYDKDAKRVGTDSVKRYNS  358

Query  61   VSVLPNTTTINGKTYYQVVENGKAVDKYINAANIDGTKRTLKHNAYVYASSKKRANKVVL  120
            VSVLPNTTTINGKTYYQVVENGKAVDKYINAANIDGTKRTLKHNAYVYASSKKRANKVVL
Sbjct  359  VSVLPNTTTINGKTYYQVVENGKAVDKYINAANIDGTKRTLKHNAYVYASSKKRANKVVL  418

Query  121  KKGEVVTTYGASYTFKNGQKYYKIGDNTDKTYVKVANFR  159
            KKGEVVTTYGASYTFKNGQKYYKIGDNTDKTYVKVANFR
Sbjct  419  KKGEVVTTYGASYTFKNGQKYYKIGDNTDKTYVKVANFR  457
```

S-layer protein [Lactobacillus acidophilus]

Sequence ID: **WP_121212587.1** Length: 457 Number of Matches: 1

Range 1: 299 to 457

| Score | Expect | Method | Identities | Positives | Gaps | Frame |
|---|---|---|---|---|---|---|
| 275 bits(702) | 2e-88() | Compositional matrix adjust. | 142/159(89%) | 145/159(91%) | 3/159(1%) | |

```
Query  4    ATSNTNGKSATLPVVVTVPN---VAEPTVASVSKRIMHNAYYYDKDAKRVGTDSVKRYNS  60
            A S  N ++A LPV V+V N   V   TV SVSKRIMHNAYYYDKDAKRVGTDSVKRYNS
Sbjct  299  AESTQNNETAQLPVTVSVTNGKEVTPSTVDSVSKRIMHNAYYYDKDAKRVGTDSVKRYNS  358

Query  61   VSVLPNTTTINGKTYYQVVENGKAVDKYINAANIDGTKRTLKHNAYVYASSKKRANKVVL  120
            VSVLPNTTTINGKTYYQVVENGKAVDKYINAANIDGTKRTLKHNAYVYASSKKRANKVVL
Sbjct  359  VSVLPNTTTINGKTYYQVVENGKAVDKYINAANIDGTKRTLKHNAYVYASSKKRANKVVL  418

Query  121  KKGEVVTTYGASYTFKNGQKYYKIGDNTDKTYVKVANFR  159
            KKGEVVTTYGASYTFKNGQKYYKIGDNTDKTYVKVANFR
Sbjct  419  KKGEVVTTYGASYTFKNGQKYYKIGDNTDKTYVKVANFR  457
```

S-layer protein, partial [Lactobacillus acidophilus]

Sequence ID: **WP_108173192.1** Length: 453 Number of Matches: 1

Range 1: 299 to 453

| Score | Expect | Method | Identities | Positives | Gaps | Frame |
|---|---|---|---|---|---|---|
| 266 bits(680) | 5e-85() | Compositional matrix adjust. | 138/155(89%) | 141/155(90%) | 3/155(1%) | |

```
Query  4    ATSNTNGKSATLPVVVTVPN---VAEPTVASVSKRIMHNAYYYDKDAKRVGTDSVKRYNS  60
            A S  N ++A LPV V+V N   V   TV SVSKRIMHNAYYYDKDAKRVGTDSVKRYNS
Sbjct  299  AESTQNNETAQLPVTVSVTNGKEVTPSTVDSVSKRIMHNAYYYDKDAKRVGTDSVKRYNS  358

Query  61   VSVLPNTTTINGKTYYQVVENGKAVDKYINAANIDGTKRTLKHNAYVYASSKKRANKVVL  120
            VSVLPNTTTINGKTYYQVVENGKAVDKYINAANIDGTKRTLKHNAYVYASSKKRANKVVL
Sbjct  359  VSVLPNTTTINGKTYYQVVENGKAVDKYINAANIDGTKRTLKHNAYVYASSKKRANKVVL  418

Query  121  KKGEVVTTYGASYTFKNGQKYYKIGDNTDKTYVKV  155
            KKGEVVTTYGASYTFKNGQKYYKIGDNTDKTYVKV
Sbjct  419  KKGEVVTTYGASYTFKNGQKYYKIGDNTDKTYVKV  453
```

S-layer protein [Lactobacillus acidophilus]

Sequence ID: **WP_025079780.1** Length: 456 Number of Matches: 1

Range 1: 298 to 456

| Score | Expect | Method | Identities | Positives | Gaps | Frame |
|---|---|---|---|---|---|---|
| 273 bits(698) | 8e-88() | Compositional matrix adjust. | 141/159(89%) | 144/159(90%) | 3/159(1%) | |

```
Query  4    ATSNTNGKSATLPVVVTVPN---VAEPTVASVSKRIMHNAYYYDKDAKRVGTDSVKRYNS  60
            A S  N ++A LPV V+V N   V   TV SVSKRIMHNAYYYDKDAKRVGTDSVKRYNS
Sbjct  298  AESTQNNETAQLPVTVSVTNGKEVTPSTVDSVSKRIMHNAYYYDKDAKRVGTDSVKRYNS  357

Query  61   VSVLPNTTTINGKTYYQVVENGKAVDKYINAANIDGTKRTLKHNAYVYASSKKRANKVVL  120
            VSVLPNTTTINGK YYQVVENGKAVDKYINAANIDGTKRTLKHNAYVYASSKKRANKVVL
Sbjct  358  VSVLPNTTTINGKAYYQVVENGKAVDKYINAANIDGTKRTLKHNAYVYASSKKRANKVVL  417

Query  121  KKGEVVTTYGASYTFKNGQKYYKIGDNTDKTYVKVANFR  159
            KKGEVVTTYGASYTFKNGQKYYKIGDNTDKTYVKVANFR
Sbjct  418  KKGEVVTTYGASYTFKNGQKYYKIGDNTDKTYVKVANFR  456
```

S-layer protein [Lactobacillus acidophilus]

Sequence ID: **WP_011254066.1** Length: 457 Number of Matches: 1

Range 1: 299 to 457

| Score | Expect | Method | Identities | Positives | Gaps | Frame |
|---|---|---|---|---|---|---|
| 273 bits(698) | 8e-88() | Compositional matrix adjust. | 141/159(89%) | 144/159(90%) | 3/159(1%) | |

```
Query  4    ATSNTNGKSATLPVVVTVPN---VAEPTVASVSKRIMHNAYYYDKDAKRVGTDSVKRYNS  60
            A S  N ++A LPV V+V N   V   TV SVSKRIMHNAYYYDKDAKRVGTDSVKRYNS
Sbjct  299  AESTQNNETAQLPVTVSVTNGKEVTPSTVDSVSKRIMHNAYYYDKDAKRVGTDSVKRYNS  358

Query  61   VSVLPNTTTINGKTYYQVVENGKAVDKYINAANIDGTKRTLKHNAYVYASSKKRANKVVL  120
            VSVLPNTTTINGK YYQVVENGKAVDKYINAANIDGTKRTLKHNAYVYASSKKRANKVVL
Sbjct  359  VSVLPNTTTINGKAYYQVVENGKAVDKYINAANIDGTKRTLKHNAYVYASSKKRANKVVL  418

Query  121  KKGEVVTTYGASYTFKNGQKYYKIGDNTDKTYVKVANFR  159
            KKGEVVTTYGASYTFKNGQKYYKIGDNTDKTYVKVANFR
Sbjct  419  KKGEVVTTYGASYTFKNGQKYYKIGDNTDKTYVKVANFR  457
```

SB-protein [Lactobacillus acidophilus]

Sequence ID: **CAA61561.1** Length: 456 Number of Matches: 1

Range 1: 298 to 456

| Score | Expect | Method | Identities | Positives | Gaps | Frame |
|---|---|---|---|---|---|---|
| 269 bits(687) | 3e-86() | Compositional matrix adjust. | 139/159(87%) | 142/159(89%) | 3/159(1%) | |

```
Query  4    ATSNTNGKSATLPVVVTVPN---VAEPTVASVSKRIMHNAYYYDKDAKRVGTDSVKRYNS  60
            A S  N ++A LPV V+V N   V   TV SVSK  MHNAYYYDKDAKRVGTDSVKRYNS
Sbjct  298  AESTQNNETAQLPVTVSVTNGKEVTPSTVDSVSKSFMHNAYYYDKDAKRVGTDSVKRYNS  357

Query  61   VSVLPNTTTINGKTYYQVVENGKAVDKYINAANIDGTKRTLKHNAYVYASSKKRANKVVL  120
            VSVLPNTTTINGK YYQVVENGKAVDKYINAANIDGTKRTLKHNAYVYASSKKRANKVVL
Sbjct  358  VSVLPNTTTINGKAYYQVVENGKAVDKYINAANIDGTKRTLKHNAYVYASSKKRANKVVL  417

Query  121  KKGEVVTTYGASYTFKNGQKYYKIGDNTDKTYVKVANFR  159
            KKGEVVTTYGASYTFKNGQKYYKIGDNTDKTYVKVANFR
Sbjct  418  KKGEVVTTYGASYTFKNGQKYYKIGDNTDKTYVKVANFR  456
```

surface layer protein [Lactobacillus helveticus]

Sequence ID: **CAB46987.1** Length: 437 Number of Matches: 1

Range 1: 278 to 437

| Score | Expect | Method | Identities | Positives | Gaps | Frame |
|---|---|---|---|---|---|---|
| 246 bits(629) | 1e-77() | Compositional matrix adjust. | 127/160(79%) | 138/160(86%) | 1/160(0%) | |

```
Query  1    NVKATSNTNGKSATLPVVVTVPNVAEPTVASVSKRIMHNAYYYDKDAKRVGTDSVKRYNS  60
            NVKATSN N KSATLPV VTVPNVAEPTV SVSK +MHNAY+YDKDAKRVGTD V RYN+
Sbjct  278  NVKATSNVNSKSATLPVTVTVPNVAEPTVPSVSKTVMHNAYFYDKDAKRVGTDKVTRYNT  337

Query  61   VSVLPNTTTI-NGKTYYQVVENGKAVDKYINAANIDGTKRTLKHNAYVYASSKKRANKVV  119
            V+V  NTT + NG +YY+V+ENGKA  KYINA NIDGTKRTLKHNAYVY +SKKRANKVV
Sbjct  338  VTVAMNTTKLANGISYYEVIENGKATGKYINADNIDGTKRTLKHNAYVYKTSKKRANKVV  397

Query  120  LKKGEVVTTYGASYTFKNGQKYYKIGDNTDKTYVKVANFR  159
            LKKG  VTTYG SY FKNGQ+YYKIG NT+KTYVKVANF
Sbjct  398  LKKGTEVTTYGGSYKFKNGQRYYKIGANTEKTYVKVANFE  437
```

S-layer protein [Lactobacillus helveticus]

Sequence ID: **AZA22401.1** Length: 442 Number of Matches: 1

Range 1: 283 to 442

| Score | Expect | Method | Identities | Positives | Gaps | Frame |
|---|---|---|---|---|---|---|
| 244 bits(623) | 1e-76() | Compositional matrix adjust. | 126/160(79%) | 138/160(86%) | 1/160(0%) | |

```
Query  1    NVKATSNTNGKSATLPVVVTVPNVAEPTVASVSKRIMHNAYYYDKDAKRVGTDSVKRYNS  60
            NVKATSN N KSATLPV VTVPNVAEPTV SVSK +MHNAY+YDK+AKRVGTD V RYN+
Sbjct  283  NVKATSNVNSKSATLPVTVTVPNVAEPTVPSVSKTVMHNAYFYDKNAKRVGTDKVTRYNT  342

Query  61   VSVLPNTTTI-NGKTYYQVVENGKAVDKYINAANIDGTKRTLKHNAYVYASSKKRANKVV  119
            V+V  NTT + NG +YY+V+ENGKA  KYINA NIDGTKRTLKHNAYVY +SKKRANKVV
Sbjct  343  VTVAMNTTKLANGISYYEVIENGKATGKYINADNIDGTKRTLKHNAYVYKTSKKRANKVV  402

Query  120  LKKGEVVTTYGASYTFKNGQKYYKIGDNTDKTYVKVANFR  159
            LKKG  VTTYG SY FKNGQ+YYKIG NT+KTYVKVANF
Sbjct  403  LKKGTEVTTYGGSYKFKNGQRYYKIGANTEKTYVKVANFE  442
```

RecName: Full=S-layer protein; AltName: Full=Surface layer protein; Flags: Precursor [Lactobacillus helveticus]

Sequence ID: **P38059.2** Length: 439 Number of Matches: 1
Range 1: 280 to 439

| Score | Expect | Method | Identities | Positives | Gaps | Frame |
|---|---|---|---|---|---|---|
| 244 bits(623) | 1e-76() | Compositional matrix adjust. | 126/160(79%) | 137/160(85%) | 1/160(0%) | |

```
Query  1     NVKATSNTNGKSATLPVVVTVPNVAEPTVASVSKRIMHNAYYYDKDAKRVGTDSVKRYNS  60
             NVKATSN NGKSATLPV VTVPNVA+P V S SK IMHNAY+YDKDAKRVGTD V RYN+
Sbjct  280   NVKATSNKNGKSATLPVTVTVPNVADPVVPSQSKTIMHNAYFYDKDAKRVGTDKVTRYNT  339

Query  61    VSVLPNTTTI-NGKTYYQVVENGKAVDKYINAANIDGTKRTLKHNAYVYASSKKRANKVV  119
             V+V  NTT + NG +YY+V+ENGKA  KYINA NIDGTKRTLKHNAYVY +SKKRANKVV
Sbjct  340   VTVAMNTTKLANGISYYEVIENGKATGKYINADNIDGTKRTLKHNAYVYKTSKKRANKVV  399

Query  120   LKKGEVVTTYGASYTFKNGQKYYKIGDNTDKTYVKVANFR  159
             LKKG  VTTYG SY FKNGQ+YYKIG NT+KTYVKVANF
Sbjct  400   LKKGTEVTTYGGSYKFKNGQRYYKIGANTEKTYVKVANFE  439
```

S-layer protein [Lactobacillus helveticus]
Sequence ID: **KGL04670.1** Length: 433 Number of Matches: 1
Range 1: 274 to 433

| Score | Expect | Method | Identities | Positives | Gaps | Frame |
|---|---|---|---|---|---|---|
| 244 bits(622) | 1e-76() | Compositional matrix adjust. | 126/160(79%) | 137/160(85%) | 1/160(0%) | |

```
Query  1     NVKATSNTNGKSATLPVVVTVPNVAEPTVASVSKRIMHNAYYYDKDAKRVGTDSVKRYNS  60
             NVKATSN NGKSATLPV VTVPNVA+P V S SK IMHNAY+YDKDAKRVGTD V RYN+
Sbjct  274   NVKATSNKNGKSATLPVTVTVPNVADPVVPSQSKTIMHNAYFYDKDAKRVGTDKVTRYNT  333

Query  61    VSVLPNTTTI-NGKTYYQVVENGKAVDKYINAANIDGTKRTLKHNAYVYASSKKRANKVV  119
             V+V  NTT + NG +YY+V+ENGKA  KYINA NIDGTKRTLKHNAYVY +SKKRANKVV
Sbjct  334   VTVAMNTTKLANGISYYEVIENGKATGKYINADNIDGTKRTLKHNAYVYKTSKKRANKVV  393

Query  120   LKKGEVVTTYGASYTFKNGQKYYKIGDNTDKTYVKVANFR  159
             LKKG  VTTYG SY FKNGQ+YYKIG NT+KTYVKVANF
Sbjct  394   LKKGTEVTTYGGSYKFKNGQRYYKIGANTEKTYVKVANFE  433
```

S-layer protein [Lactobacillus helveticus]
Sequence ID: **WP_095662016.1** Length: 439 Number of Matches: 1
Range 1: 280 to 439

| Score | Expect | Method | Identities | Positives | Gaps | Frame |
|---|---|---|---|---|---|---|
| 244 bits(622) | 1e-76() | Compositional matrix adjust. | 126/160(79%) | 137/160(85%) | 1/160(0%) | |

```
Query  1     NVKATSNTNGKSATLPVVVTVPNVAEPTVASVSKRIMHNAYYYDKDAKRVGTDSVKRYNS  60
             NVKATSN NGKSATLPV VTVPNVA+P V S SK IMHNAY+YDKDAKRVGTD V RYN+
Sbjct  280   NVKATSNKNGKSATLPVTVTVPNVADPVVPSQSKTIMHNAYFYDKDAKRVGTDKVTRYNT  339

Query  61    VSVLPNTTTI-NGKTYYQVVENGKAVDKYINAANIDGTKRTLKHNAYVYASSKKRANKVV  119
             V+V  NTT + NG +YY+V+ENGKA  KYINA NIDGTKRTLKHNAYVY +SKKRANKVV
Sbjct  340   VTVAMNTTKLANGISYYEVIENGKATGKYINADNIDGTKRTLKHNAYVYKTSKKRANKVV  399

Query  120   LKKGEVVTTYGASYTFKNGQKYYKIGDNTDKTYVKVANFR  159
             LKKG  VTTYG SY FKNGQ+YYKIG NT+KTYVKVANF
Sbjct  400   LKKGTEVTTYGGSYKFKNGQRYYKIGANTEKTYVKVANFE  439
```

surface layer protein [Lactobacillus helveticus]
Sequence ID: **ASS83148.1** Length: 442 Number of Matches: 1
Range 1: 283 to 442

| Score | Expect | Method | Identities | Positives | Gaps | Frame |
|---|---|---|---|---|---|---|
| 244 bits(623) | 1e-76() | Compositional matrix adjust. | 126/160(79%) | 138/160(86%) | 1/160(0%) | |

```
Query  1     NVKATSNTNGKSATLPVVVTVPNVAEPTVASVSKRIMHNAYYYDKDAKRVGTDSVKRYNS  60
             NVKATSN N KSATLPV VTVPNVAEPTV SVSK +MHNAY+YDK+AKRVGTD V RYN+
Sbjct  283   NVKATSNVNSKSATLPVTVTVPNVAEPTVPSVSKTVMHNAYFYDKNAKRVGTDKVTRYNT  342

Query  61    VSVLPNTTTI-NGKTYYQVVENGKAVDKYINAANIDGTKRTLKHNAYVYASSKKRANKVV  119
             V+V  NTT + NG +YY+V+ENGKA  KYINA NIDGTKRTLKHNAYVY +SKKRANKVV
Sbjct  343   VTVAMNTTKLANGISYYEVIENGKATGKYINADNIDGTKRTLKHNAYVYKTSKKRANKVV  402

Query  120   LKKGEVVTTYGASYTFKNGQKYYKIGDNTDKTYVKVANFR  159
             LKKG  VTTYG SY FKNGQ+YYKIG NT+KTYVKVANF
Sbjct  403   LKKGTEVTTYGGSYKFKNGQRYYKIGANTEKTYVKVANFE  442
```

surface layer protein [Lactobacillus helveticus]
Sequence ID: **CAA63409.1** Length: 439 Number of Matches: 1
Range 1: 280 to 439

| Score | Expect | Method | Identities | Positives | Gaps | Frame |
|---|---|---|---|---|---|---|

| | | | | | | |
|---|---|---|---|---|---|---|
| 244 bits(622) | 1e-76() | Compositional matrix adjust. | 126/160(79%) | 137/160(85%) | 1/160(0%) | |

```
Query  1    NVKATSNTNGKSATLPVVVTVPNVAEPTVASVSKRIMHNAYYYDKDAKRVGTDSVKRYNS  60
            NVKATSN NGKSATLPV VTVPNVA+P V S SK IMHNAY+YDKDAKRVGTD V RYN+
Sbjct  280  NVKATSNKNGKSATLPVTVTVPNVADPVVPSQSKTIMHNAYFYDKDAKRVGTDKVTRYNT  339

Query  61   VSVLPNTTTI-NGKTYYQVVENGKAVDKYINAANIDGTKRTLKHNAYVYASSKKRANKVV  119
            V+V  NTT + NG +YY+V+ENGKA  KYINA NIDGTKRTLKHNAYVY +SKKRANKVV
Sbjct  340  VTVAMNTTKLANGISYYEVIENGKATGKYINADNIDGTKRTLKHNAYVYKTSKKRANKVV  399

Query  120  LKKGEVVTTYGASYTFKNGQKYYKIGDNTDKTYVKVANFR  159
            LKKG  VTTYG SY FKNGQ+YYKIG NT+KTYVKVANF
Sbjct  400  LKKGTEVTTYGGSYKFKNGQRYYKIGANTEKTYVKVANFE  439
```

surface layer protein [Lactobacillus helveticus]

Sequence ID: **CAB46986.1** Length: 439 Number of Matches: 1
Range 1: 280 to 439

| Score | Expect | Method | Identities | Positives | Gaps | Frame |
|---|---|---|---|---|---|---|
| 244 bits(622) | 1e-76() | Compositional matrix adjust. | 126/160(79%) | 137/160(85%) | 1/160(0%) | |

```
Query  1    NVKATSNTNGKSATLPVVVTVPNVAEPTVASVSKRIMHNAYYYDKDAKRVGTDSVKRYNS  60
            NVKATSN NGKSATLPV VTVPNVA+P V S SK IMHNAY+YDKDAKRVGTD V RYN+
Sbjct  280  NVKATSNKNGKSATLPVTVTVPNVADPVVPSQSKTIMHNAYFYDKDAKRVGTDKVTRYNT  339

Query  61   VSVLPNTTTI-NGKTYYQVVENGKAVDKYINAANIDGTKRTLKHNAYVYASSKKRANKVV  119
            V+V  NTT + NG +YY+V+ENGKA  KYINA NIDGTKRTLKHNAYVY +SKKRANKVV
Sbjct  340  VTVAMNTTKLANGISYYEVIENGKATGKYINADNIDGTKRTLKHNAYVYKTSKKRANKVV  399

Query  120  LKKGEVVTTYGASYTFKNGQKYYKIGDNTDKTYVKVANFR  159
            LKKG  VTTYG SY FKNGQ+YYKIG NT+KTYVKVANF
Sbjct  400  LKKGTEVTTYGGSYKFKNGQRYYKIGANTEKTYVKVANFE  439
```

S-layer protein [Lactobacillus helveticus]

Sequence ID: **WP_097550829.1** Length: 439 Number of Matches: 1
Range 1: 280 to 439

| Score | Expect | Method | Identities | Positives | Gaps | Frame |
|---|---|---|---|---|---|---|
| 244 bits(622) | 1e-76() | Compositional matrix adjust. | 126/160(79%) | 137/160(85%) | 1/160(0%) | |

```
Query  1    NVKATSNTNGKSATLPVVVTVPNVAEPTVASVSKRIMHNAYYYDKDAKRVGTDSVKRYNS  60
            NVKATSN NGKSATLPV VTVPNVA+P V S SK IMHNAY+YDKDAKRVGTD V RYN+
Sbjct  280  NVKATSNKNGKSATLPVTVTVPNVADPVVPSQSKTIMHNAYFYDKDAKRVGTDKVTRYNT  339

Query  61   VSVLPNTTTI-NGKTYYQVVENGKAVDKYINAANIDGTKRTLKHNAYVYASSKKRANKVV  119
            V+V  NTT + NG +YY+V+ENGKA  KYINA NIDGTKRTLKHNAYVY +SKKRANKVV
Sbjct  340  VTVAMNTTKLANGISYYEVIENGKATGKYINADNIDGTKRTLKHNAYVYKTSKKRANKVV  399

Query  120  LKKGEVVTTYGASYTFKNGQKYYKIGDNTDKTYVKVANFR  159
            LKKG  VTTYG SY FKNGQ+YYKIG NT+KTYVKVANF
Sbjct  400  LKKGTEVTTYGGSYKFKNGQRYYKIGANTEKTYVKVANFE  439
```

surface layer protein [Lactobacillus helveticus DSM 20075 = CGMCC 1.1877]

Sequence ID: **CAB46985.1** Length: 439 Number of Matches: 1
Range 1: 280 to 439

| Score | Expect | Method | Identities | Positives | Gaps | Frame |
|---|---|---|---|---|---|---|
| 244 bits(622) | 1e-76() | Compositional matrix adjust. | 126/160(79%) | 137/160(85%) | 1/160(0%) | |

```
Query  1    NVKATSNTNGKSATLPVVVTVPNVAEPTVASVSKRIMHNAYYYDKDAKRVGTDSVKRYNS  60
            NVKATSN NGKSATLPV VTVPNVA+P V S SK IMHNAY+YDKDAKRVGTD V RYN+
Sbjct  280  NVKATSNKNGKSATLPVTVTVPNVADPVVPSQSKTIMHNAYFYDKDAKRVGTDKVTRYNT  339

Query  61   VSVLPNTTTI-NGKTYYQVVENGKAVDKYINAANIDGTKRTLKHNAYVYASSKKRANKVV  119
            V+V  NTT + NG +YY+V+ENGKA  KYINA NIDGTKRTLKHNAYVY +SKKRANKVV
Sbjct  340  VTVAMNTTKLANGISYYEVIENGKATGKYINADNIDGTKRTLKHNAYVYKTSKKRANKVV  399

Query  120  LKKGEVVTTYGASYTFKNGQKYYKIGDNTDKTYVKVANFR  159
            LKKG  VTTYG SY FKNGQ+YYKIG NT+KTYVKVANF
Sbjct  400  LKKGTEVTTYGGSYKFKNGQRYYKIGANTEKTYVKVANFE  439
```

surface layer protein [Lactobacillus helveticus]

Sequence ID: **CAB46988.1** Length: 439 Number of Matches: 1
Range 1: 280 to 439

| Score | Expect | Method | Identities | Positives | Gaps | Frame |
|---|---|---|---|---|---|---|
| 244 bits(622) | 2e-76() | Compositional matrix adjust. | 126/160(79%) | 137/160(85%) | 1/160(0%) | |

```
Query  1    NVKATSNTNGKSATLPVVVTVPNVAEPTVASVSKRIMHNAYYYDKDAKRVGTDSVKRYNS  60
            NVKATSN NGKSATLPV VTVPNVA+P V S SK IMHNAY+YDKDAKRVGTD V RYN+
Sbjct  280  NVKATSNKNGKSATLPVTVTVPNVADPVVPSQSKTIMHNAYFYDKDAKRVGTDKVTRYNT  339
```

```
Query  61   VSVLPNTTTI-NGKTYYQVVENGKAVDKYINAANIDGTKRTLKHNAYVYASSKKRANKVV  119
            V+V  NTT + NG +YY+V+ENGKA  KYINA NIDGTKRTLKHNAYVY +SKKRANKVV
Sbjct  340  VTVAMNTTKLANGISYYEVIENGKATGKYINADNIDGTKRTLKHNAYVYKTSKKRANKVV  399

Query  120  LKKGEVVTTYGASYTFKNGQKYYKIGDNTDKTYVKVANFR  159
            LKKG  VTTYG SY FKNGQ+YYKIG NT+KTYVKVANF
Sbjct  400  LKKGTEVTTYGGSYKFKNGQRYYKIGANTEKTYVKVANFE  439
```

S-layer protein [Lactobacillus helveticus]

Sequence ID: **WP_110554553.1** Length: 439 Number of Matches: 1

Range 1: 280 to 439

| Score | Expect | Method | Identities | Positives | Gaps | Frame |
|---|---|---|---|---|---|---|
| 244 bits(622) | 2e-76() | Compositional matrix adjust. | 126/160(79%) | 137/160(85%) | 1/160(0%) | |

```
Query  1    NVKATSNTNGKSATLPVVVTVPNVAEPTVASVSKRIMHNAYYYDKDAKRVGTDSVKRYNS  60
            NVKATSN NGKSATLPV VTVPNVA+P V S SK IMHNAY+YDKDAKRVGTD V RYN+
Sbjct  280  NVKATSNKNGKSATLPVTVTVPNVADPVVPSQSKTIMHNAYFYDKDAKRVGTDKVTRYNT  339

Query  61   VSVLPNTTTI-NGKTYYQVVENGKAVDKYINAANIDGTKRTLKHNAYVYASSKKRANKVV  119
            V+V  NTT + NG +YY+V+ENGKA  KYINA NIDGTKRTLKHNAYVY +SKKRANKVV
Sbjct  340  VTVAMNTTKLANGISYYEVIENGKATGKYINADNIDGTKRTLKHNAYVYKTSKKRANKVV  399

Query  120  LKKGEVVTTYGASYTFKNGQKYYKIGDNTDKTYVKVANFR  159
            LKKG  VTTYG SY FKNGQ+YYKIG NT+KTYVKVANF
Sbjct  400  LKKGTEVTTYGGSYKFKNGQRYYKIGANTEKTYVKVANFE  439
```

S-layer protein [Lactobacillus helveticus]

Sequence ID: **KXN79916.1** Length: 440 Number of Matches: 1

Range 1: 281 to 440

| Score | Expect | Method | Identities | Positives | Gaps | Frame |
|---|---|---|---|---|---|---|
| 244 bits(622) | 2e-76() | Compositional matrix adjust. | 126/160(79%) | 137/160(85%) | 1/160(0%) | |

```
Query  1    NVKATSNTNGKSATLPVVVTVPNVAEPTVASVSKRIMHNAYYYDKDAKRVGTDSVKRYNS  60
            NVKATSN NGKSATLPV VTVPNVA+P V S SK IMHNAY+YDKDAKRVGTD V RYN+
Sbjct  281  NVKATSNKNGKSATLPVTVTVPNVADPVVPSQSKTIMHNAYFYDKDAKRVGTDKVTRYNT  340

Query  61   VSVLPNTTTI-NGKTYYQVVENGKAVDKYINAANIDGTKRTLKHNAYVYASSKKRANKVV  119
            V+V  NTT + NG +YY+V+ENGKA  KYINA NIDGTKRTLKHNAYVY +SKKRANKVV
Sbjct  341  VTVAMNTTKLANGISYYEVIENGKATGKYINADNIDGTKRTLKHNAYVYKTSKKRANKVV  400

Query  120  LKKGEVVTTYGASYTFKNGQKYYKIGDNTDKTYVKVANFR  159
            LKKG  VTTYG SY FKNGQ+YYKIG NT+KTYVKVANF
Sbjct  401  LKKGTEVTTYGGSYKFKNGQRYYKIGANTEKTYVKVANFE  440
```

S-layer protein [Lactobacillus helveticus]

Sequence ID: **WP_110534788.1** Length: 439 Number of Matches: 1

Range 1: 280 to 439

| Score | Expect | Method | Identities | Positives | Gaps | Frame |
|---|---|---|---|---|---|---|
| 244 bits(622) | 2e-76() | Compositional matrix adjust. | 126/160(79%) | 137/160(85%) | 1/160(0%) | |

```
Query  1    NVKATSNTNGKSATLPVVVTVPNVAEPTVASVSKRIMHNAYYYDKDAKRVGTDSVKRYNS  60
            NVKATSN NGKSATLPV VTVPNVA+P V S SK IMHNAY+YDKDAKRVGTD V RYN+
Sbjct  280  NVKATSNKNGKSATLPVTVTVPNVADPVVPSQSKTIMHNAYFYDKDAKRVGTDKVTRYNT  339

Query  61   VSVLPNTTTI-NGKTYYQVVENGKAVDKYINAANIDGTKRTLKHNAYVYASSKKRANKVV  119
            V+V  NTT + NG +YY+V+ENGKA  KYINA NIDGTKRTLKHNAYVY +SKKRANKVV
Sbjct  340  VTVAMNTTKLANGISYYEVIENGKATGKYINADNIDGTKRTLKHNAYVYKTSKKRANKVV  399

Query  120  LKKGEVVTTYGASYTFKNGQKYYKIGDNTDKTYVKVANFR  159
            LKKG  VTTYG SY FKNGQ+YYKIG NT+KTYVKVANF
Sbjct  400  LKKGTEVTTYGGSYKFKNGQRYYKIGANTEKTYVKVANFE  439
```

surface layer protein [Lactobacillus helveticus]

Sequence ID: **ASS83145.1** Length: 439 Number of Matches: 1

Range 1: 280 to 439

| Score | Expect | Method | Identities | Positives | Gaps | Frame |
|---|---|---|---|---|---|---|
| 244 bits(622) | 2e-76() | Compositional matrix adjust. | 126/160(79%) | 137/160(85%) | 1/160(0%) | |

```
Query  1    NVKATSNTNGKSATLPVVVTVPNVAEPTVASVSKRIMHNAYYYDKDAKRVGTDSVKRYNS  60
            NVKATSN NGKSATLPV VTVPNVA+P V S SK IMHNAY+YDKDAKRVGTD V RYN+
Sbjct  280  NVKATSNKNGKSATLPVTVTVPNVADPVVPSQSKTIMHNAYFYDKDAKRVGTDKVTRYNT  339

Query  61   VSVLPNTTTI-NGKTYYQVVENGKAVDKYINAANIDGTKRTLKHNAYVYASSKKRANKVV  119
            V+V  NTT + NG +YY+V+ENGKA  KYINA NIDGTKRTLKHNAYVY +SKKRANKVV
Sbjct  340  VTVAMNTTKLANGISYYEVIENGKATGKYINADNIDGTKRTLKHNAYVYKTSKKRANKVV  399

Query  120  LKKGEVVTTYGASYTFKNGQKYYKIGDNTDKTYVKVANFR  159
            LKKG  VTTYG SY FKNGQ+YYKIG NT+KTYVKVANF
Sbjct  400  LKKGTEVTTYGGSYKFKNGQRYYKIGANTEKTYVKVANFE  439
```

S-layer protein [Lactobacillus helveticus]

Sequence ID: **WP_080742792.1** Length: 466 Number of Matches: 1

Range 1: 307 to 466

| Score | Expect | Method | Identities | Positives | Gaps | Frame |
|---|---|---|---|---|---|---|
| 243 bits(621) | 4e-76() | Compositional matrix adjust. | 126/160(79%) | 137/160(85%) | 1/160(0%) | |

```
Query  1    NVKATSNTNGKSATLPVVVTVPNVAEPTVASVSKRIMHNAYYYDKDAKRVGTDSVKRYNS  60
            NVKATSN NGKSATLPV VTVPNVA+P V S SK IMHNAY+YDKDAKRVGTD V RYN+
Sbjct  307  NVKATSNKNGKSATLPVTVTVPNVADPVVPSQSKTIMHNAYFYDKDAKRVGTDKVTRYNT  366

Query  61   VSVLPNTTTI-NGKTYYQVVENGKAVDKYINAANIDGTKRTLKHNAYVYASSKKRANKVV  119
            V+V  NTT + NG +YY+V+ENGKA  KYINA NIDGTKRTLKHNAYVY +SKKRANKVV
Sbjct  367  VTVAMNTTKLANGISYYEVIENGKATGKYINADNIDGTKRTLKHNAYVYKTSKKRANKVV  426

Query  120  LKKGEVVTTYGASYTFKNGQKYYKIGDNTDKTYVKVANFR  159
            LKKG  VTTYG SY FKNGQ+YYKIG NT+KTYVKVANF
Sbjct  427  LKKGTEVTTYGGSYKFKNGQRYYKIGANTEKTYVKVANFE  466
```

S-layer protein [Lactobacillus helveticus]

Sequence ID: **WP_080669082.1** Length: 473 Number of Matches: 1

Range 1: 314 to 473

| Score | Expect | Method | Identities | Positives | Gaps | Frame |
|---|---|---|---|---|---|---|
| 244 bits(622) | 4e-76() | Compositional matrix adjust. | 126/160(79%) | 137/160(85%) | 1/160(0%) | |

```
Query  1    NVKATSNTNGKSATLPVVVTVPNVAEPTVASVSKRIMHNAYYYDKDAKRVGTDSVKRYNS  60
            NVKATSN NGKSATLPV VTVPNVA+P V S SK IMHNAY+YDKDAKRVGTD V RYN+
Sbjct  314  NVKATSNKNGKSATLPVTVTVPNVADPVVPSQSKTIMHNAYFYDKDAKRVGTDKVTRYNT  373

Query  61   VSVLPNTTTI-NGKTYYQVVENGKAVDKYINAANIDGTKRTLKHNAYVYASSKKRANKVV  119
            V+V  NTT + NG +YY+V+ENGKA  KYINA NIDGTKRTLKHNAYVY +SKKRANKVV
Sbjct  374  VTVAMNTTKLANGISYYEVIENGKATGKYINADNIDGTKRTLKHNAYVYKTSKKRANKVV  433

Query  120  LKKGEVVTTYGASYTFKNGQKYYKIGDNTDKTYVKVANFR  159
            LKKG  VTTYG SY FKNGQ+YYKIG NT+KTYVKVANF
Sbjct  434  LKKGTEVTTYGGSYKFKNGQRYYKIGANTEKTYVKVANFE  473
```

S-layer protein [Lactobacillus helveticus]

Sequence ID: **WP_003625506.1** Length: 472 Number of Matches: 1

Range 1: 313 to 472

| Score | Expect | Method | Identities | Positives | Gaps | Frame |
|---|---|---|---|---|---|---|
| 244 bits(622) | 4e-76() | Compositional matrix adjust. | 126/160(79%) | 137/160(85%) | 1/160(0%) | |

```
Query  1    NVKATSNTNGKSATLPVVVTVPNVAEPTVASVSKRIMHNAYYYDKDAKRVGTDSVKRYNS  60
            NVKATSN NGKSATLPV VTVPNVA+P V S SK IMHNAY+YDKDAKRVGTD V RYN+
Sbjct  313  NVKATSNKNGKSATLPVTVTVPNVADPVVPSQSKTIMHNAYFYDKDAKRVGTDKVTRYNT  372

Query  61   VSVLPNTTTI-NGKTYYQVVENGKAVDKYINAANIDGTKRTLKHNAYVYASSKKRANKVV  119
            V+V  NTT + NG +YY+V+ENGKA  KYINA NIDGTKRTLKHNAYVY +SKKRANKVV
Sbjct  373  VTVAMNTTKLANGISYYEVIENGKATGKYINADNIDGTKRTLKHNAYVYKTSKKRANKVV  432

Query  120  LKKGEVVTTYGASYTFKNGQKYYKIGDNTDKTYVKVANFR  159
            LKKG  VTTYG SY FKNGQ+YYKIG NT+KTYVKVANF
Sbjct  433  LKKGTEVTTYGGSYKFKNGQRYYKIGANTEKTYVKVANFE  472
```

## Taxonomy

### Reports

### Lineage

| Organism | Blast Name | Score | Number of Hits | Description |
|---|---|---|---|---|
| Lactobacillus | firmicutes | | 126 | |
| .Lactobacillus acidophilus | firmicutes | 318 | 31 | **Lactobacillus acidophilus hits** |
| .Lactobacillus acidophilus NCFM | firmicutes | 317 | 5 | **Lactobacillus acidophilus NCFM hits** |
| .Lactobacillus acidophilus La-14 | firmicutes | 317 | 2 | **Lactobacillus acidophilus La-14 hits** |
| .Lactobacillus acidophilus DSM 20242 | firmicutes | 316 | 2 | **Lactobacillus acidophilus DSM 20242 hits** |
| .Lactobacillus acidophilus CIRM-BIA 442 | firmicutes | 315 | 2 | **Lactobacillus acidophilus CIRM-BIA 442 hits** |
| .Lactobacillus helveticus | firmicutes | 246 | 50 | **Lactobacillus helveticus hits** |

| | | | | |
|---|---|---|---|---|
| .Lactobacillus helveticus DSM 20075 = CGMCC 1.1877 | firmicutes | 244 | 3 | Lactobacillus helveticus DSM 20075 = CGMCC 1.1877 hits |
| .Lactobacillus helveticus CIRM-BIA 101 | firmicutes | 244 | 1 | Lactobacillus helveticus CIRM-BIA 101 hits |
| .Lactobacillus helveticus CIRM-BIA 104 | firmicutes | 244 | 1 | Lactobacillus helveticus CIRM-BIA 104 hits |
| .Lactobacillus kefiranofaciens | firmicutes | 243 | 2 | Lactobacillus kefiranofaciens hits |
| .Lactobacillus kefiranofaciens ZW3 | firmicutes | 243 | 1 | Lactobacillus kefiranofaciens ZW3 hits |
| .Lactobacillus kefiranofaciens subsp. kefiranofaciens DSM 5016 = JCM 6985 | firmicutes | 243 | 1 | Lactobacillus kefiranofaciens subsp. kefiranofaciens DSM 5016 = JCM 6985 hits |
| .Lactobacillus amylovorus | firmicutes | 242 | 8 | Lactobacillus amylovorus hits |
| .Lactobacillus amylovorus DSM 20531 | firmicutes | 242 | 2 | Lactobacillus amylovorus DSM 20531 hits |
| .Lactobacillus helveticus CIRM-BIA 953 | firmicutes | 241 | 2 | Lactobacillus helveticus CIRM-BIA 953 hits |
| .Lactobacillus gallinarum | firmicutes | 236 | 3 | Lactobacillus gallinarum hits |
| .Lactobacillus gallinarum DSM 10532 = JCM 2011 | firmicutes | 236 | 1 | Lactobacillus gallinarum DSM 10532 = JCM 2011 hits |
| .Lactobacillus helveticus H10 | firmicutes | 236 | 1 | Lactobacillus helveticus H10 hits |
| .Lactobacillus crispatus | firmicutes | 233 | 5 | Lactobacillus crispatus hits |
| .Lactobacillus amylovorus DSM 16698 | firmicutes | 233 | 1 | Lactobacillus amylovorus DSM 16698 hits |
| .Lactobacillus amylovorus GRL1118 | firmicutes | 232 | 1 | Lactobacillus amylovorus GRL1118 hits |
| .Lactobacillus helveticus DPC 4571 | firmicutes | 224 | 1 | Lactobacillus helveticus DPC 4571 hits |

## Organism

○

| Description | Score | E value | Accession |
|---|---|---|---|
| Lactobacillus acidophilus [firmicutes ] | | | |
| S-layer protein [Lactobacillus acidophilus] | 318 | 2e-105 | WP_125977721 |
| S-layer protein [Lactobacillus acidophilus] | 318 | 2e-105 | AZN75822 |
| S-layer protein [Lactobacillus acidophilus] | 317 | 2e-105 | WP_075917333 |
| S-layer protein [Lactobacillus acidophilus] | 317 | 3e-105 | WP_089541165 |
| S-layer protein [Lactobacillus acidophilus] | 317 | 3e-105 | ASN46111 |
| S-layer protein [Lactobacillus acidophilus] | 317 | 3e-105 | WP_011254065 |
| principal surface-layer protein SlpA/SINGR3-dependent intestinal immunomodulator [Lactobacillus acidophilus] | 317 | 3e-105 | AJP45643 |
| S-layer protein [Lactobacillus acidophilus] | 317 | 3e-105 | ASX14185 |
| S-layer protein [Lactobacillus acidophilus] | 317 | 3e-105 | AVW87822 |
| S-layer protein [Lactobacillus acidophilus] | 316 | 1e-104 | WP_021721641 |
| S-layer protein [Lactobacillus acidophilus] | 315 | 2e-104 | WP_021721262 |
| S-layer protein [Lactobacillus acidophilus] | 315 | 2e-104 | PTS31834 |
| S-layer protein [Lactobacillus acidophilus] | 315 | 2e-104 | PTS68287 |
| S-layer protein, partial [Lactobacillus acidophilus] | 306 | 3e-101 | WP_063720662 |
| S-layer protein, partial [Lactobacillus acidophilus] | 306 | 3e-101 | KZX18392 |
| surface layer protein [Lactobacillus acidophilus] | 277 | 3e-89 | AEW12794 |
| S-layer protein, partial [Lactobacillus acidophilus] | 274 | 8e-89 | WP_042710617 |
| S-layer protein [Lactobacillus acidophilus] | 275 | 1e-88 | WP_125977729 |
| S-layer protein [Lactobacillus acidophilus] | 275 | 1e-88 | AZN75826 |
| S-layer protein [Lactobacillus acidophilus] | 275 | 2e-88 | WP_121212587 |
| S-layer protein [Lactobacillus acidophilus] | 275 | 2e-88 | RKN32723 |
| S-layer protein [Lactobacillus acidophilus] | 275 | 2e-88 | TDB30449 |
| S-layer protein [Lactobacillus acidophilus] | 273 | 8e-88 | WP_025079780 |
| S-layer protein [Lactobacillus acidophilus] | 273 | 8e-88 | AVW87825 |
| S-layer protein [Lactobacillus acidophilus] | 273 | 8e-88 | WP_011254066 |
| auxiliary surface-layer protein SlpB [Lactobacillus acidophilus] | 273 | 8e-88 | AJP45647 |
| SB-protein [Lactobacillus acidophilus] | 269 | 3e-86 | CAA61561 |
| S-layer protein, partial [Lactobacillus acidophilus] | 266 | 5e-85 | WP_108173192 |

| Description | Score | E value | Accession |
|---|---|---|---|
| S-layer protein, partial [Lactobacillus acidophilus] | 266 | 5e-85 | PTV30052 |
| S-layer protein, partial [Lactobacillus acidophilus] | 241 | 9e-80 | PTV27709 |
| S-layer protein, partial [Lactobacillus acidophilus] | 236 | 3e-74 | WP_029779823 |
| Lactobacillus acidophilus NCFM [firmicutes] | | | |
| S-layer protein [Lactobacillus acidophilus NCFM] | 317 | 3e-105 | YP_193101 |
| RecName: Full=S-layer protein; AltName: Full=Surface layer protein; Short=SA-protein; Flags: Precursor [Lactobacillus acidophilus NCFM] | 317 | 3e-105 | P35829 |
| S-layer [Lactobacillus acidophilus NCFM] | 317 | 3e-105 | AAV42070 |
| S-layer protein [Lactobacillus acidophilus NCFM] | 273 | 8e-88 | YP_193105 |
| S-layer [Lactobacillus acidophilus NCFM] | 273 | 8e-88 | AAV42074 |
| Lactobacillus acidophilus La-14 [firmicutes] | | | |
| S-layer protein precursor [Lactobacillus acidophilus La-14] | 317 | 3e-105 | AGK93397 |
| S-layer protein precursor [Lactobacillus acidophilus La-14] | 273 | 8e-88 | AGK93401 |
| Lactobacillus acidophilus DSM 20242 [firmicutes] | | | |
| S-layer protein [Lactobacillus acidophilus DSM 20242] | 316 | 1e-104 | CDF76236 |
| S-layer [Lactobacillus acidophilus DSM 20242] | 273 | 8e-88 | CDF76232 |
| Lactobacillus acidophilus CIRM-BIA 442 [firmicutes] | | | |
| S-layer protein [Lactobacillus acidophilus CIRM-BIA 442] | 315 | 2e-104 | CDF68649 |
| S-layer [Lactobacillus acidophilus CIRM-BIA 442] | 273 | 8e-88 | CDF68653 |
| Lactobacillus helveticus [firmicutes] | | | |
| surface layer protein [Lactobacillus helveticus] | 246 | 1e-77 | CAB46987 |
| S-layer protein [Lactobacillus helveticus] | 244 | 1e-76 | AZA22401 |
| RecName: Full=S-layer protein; AltName: Full=Surface layer protein; Flags: Precursor [Lactobacillus helveticus] | 244 | 1e-76 | P38059 |
| S-layer protein [Lactobacillus helveticus] | 244 | 1e-76 | KGL06356 |
| surface layer protein [Lactobacillus helveticus] | 244 | 1e-76 | CAA62606 |
| S-layer protein [Lactobacillus helveticus] | 244 | 1e-76 | KGL04670 |
| S-layer protein [Lactobacillus helveticus] | 244 | 1e-76 | WP_095662016 |
| S-layer protein [Lactobacillus helveticus] | 244 | 1e-76 | PAW07148 |
| surface layer protein [Lactobacillus helveticus] | 244 | 1e-76 | ASS83148 |
| surface layer protein [Lactobacillus helveticus] | 244 | 1e-76 | CAA63409 |
| surface layer protein [Lactobacillus helveticus] | 244 | 1e-76 | CAB46986 |
| S-layer protein [Lactobacillus helveticus] | 244 | 1e-76 | WP_097550829 |
| surface layer protein [Lactobacillus helveticus] | 244 | 2e-76 | CAB46988 |
| S-layer protein [Lactobacillus helveticus] | 244 | 2e-76 | WP_110554553 |
| surface layer protein [Lactobacillus helveticus] | 244 | 2e-76 | ASS83144 |
| S-layer protein [Lactobacillus helveticus] | 244 | 2e-76 | PXZ20362 |
| S-layer protein [Lactobacillus helveticus] | 244 | 2e-76 | KXN79916 |
| S-layer protein [Lactobacillus helveticus] | 244 | 2e-76 | WP_110534788 |
| surface layer protein [Lactobacillus helveticus] | 244 | 2e-76 | ASS83146 |
| S-layer protein [Lactobacillus helveticus] | 244 | 2e-76 | PXZ21460 |
| surface layer protein [Lactobacillus helveticus] | 244 | 2e-76 | ASS83145 |
| surface layer protein [Lactobacillus helveticus] | 244 | 2e-76 | CAB46984 |
| surface layer protein [Lactobacillus helveticus] | 243 | 2e-76 | CAB46990 |
| S-layer protein [Lactobacillus helveticus] | 243 | 4e-76 | WP_080742792 |
| S-layer protein [Lactobacillus helveticus] | 244 | 4e-76 | WP_080669082 |
| S-layer protein [Lactobacillus helveticus] | 244 | 4e-76 | WP_003625506 |
| surface layer protein [Lactobacillus helveticus] | 242 | 7e-76 | CAB46989 |
| surface layer protein [Lactobacillus helveticus] | 241 | 2e-75 | WP_023061216 |
| S-layer protein [Lactobacillus helveticus] | 241 | 2e-75 | ALI51846 |
| S-layer protein [Lactobacillus helveticus] | 239 | 2e-74 | WP_080669035 |

| Description | Score | E value | Accession |
|---|---|---|---|
| S-layer protein [Lactobacillus helveticus] | 236 | 1e-73 | WP_041809302 |
| S-layer protein [Lactobacillus helveticus] | 234 | 5e-73 | WP_101511756 |
| surface layer protein [Lactobacillus helveticus] | 234 | 5e-73 | ASS83147 |
| Silent surface layer protein SlpA (Fragment) [Lactobacillus helveticus] | 234 | 5e-73 | SPS14145 |
| S-layer protein [Lactobacillus helveticus] | 230 | 4e-71 | WP_046813800 |
| S-layer protein [Lactobacillus helveticus] | 230 | 4e-71 | AKG66052 |
| surface layer protein [Lactobacillus helveticus] | 229 | 9e-71 | KRO14174 |
| hypothetical protein [Lactobacillus helveticus] | 224 | 5e-69 | WP_041810676 |
| S-layer protein [Lactobacillus helveticus] | 224 | 5e-69 | WP_111194039 |
| surface layer protein [Lactobacillus helveticus] | 224 | 5e-69 | ASS83157 |
| S-layer protein [Lactobacillus helveticus] | 224 | 5e-69 | PZD75967 |
| S-layer protein [Lactobacillus helveticus] | 224 | 5e-69 | WP_101853699 |
| surface layer protein [Lactobacillus helveticus] | 224 | 5e-69 | ASS83156 |
| hypothetical protein Lh8105_00915 [Lactobacillus helveticus] | 224 | 5e-69 | AUI73557 |
| S-layer protein [Lactobacillus helveticus] | 224 | 5e-69 | PXZ14395 |
| S-layer protein [Lactobacillus helveticus] | 224 | 5e-69 | PXZ16236 |
| S-layer protein [Lactobacillus helveticus] | 224 | 5e-69 | PXZ23386 |
| surface layer protein [Lactobacillus helveticus] | 224 | 6e-69 | ASS83158 |
| S-layer protein [Lactobacillus helveticus] | 224 | 6e-69 | AZA19251 |
| hypothetical protein [Lactobacillus helveticus] | 224 | 6e-69 | WP_072749112 |
| Lactobacillus helveticus DSM 20075 = CGMCC 1.1877 [firmicutes ] | | | |
| hypothetical protein FC11_GL001610 [Lactobacillus helveticus DSM 20075 = CGMCC 1.1877] | 244 | 1e-76 | KRL33149 |
| surface layer protein [Lactobacillus helveticus DSM 20075 = CGMCC 1.1877] | 244 | 1e-76 | CAB46985 |
| bacterial surface layer protein [Lactobacillus helveticus DSM 20075 = CGMCC 1.1877] | 244 | 4e-76 | EEW68127 |
| Lactobacillus helveticus CIRM-BIA 101 [firmicutes ] | | | |
| Surface layer protein [Lactobacillus helveticus CIRM-BIA 101] | 244 | 1e-76 | CDI65858 |
| Lactobacillus helveticus CIRM-BIA 104 [firmicutes ] | | | |
| Surface layer protein [Lactobacillus helveticus CIRM-BIA 104] | 244 | 2e-76 | CDI59467 |
| Lactobacillus kefiranofaciens [firmicutes ] | | | |
| S-layer protein [Lactobacillus kefiranofaciens] | 243 | 5e-76 | WP_013855236 |
| surface layer protein [Lactobacillus kefiranofaciens] | 243 | 5e-76 | SDA65752 |
| Lactobacillus kefiranofaciens ZW3 [firmicutes ] | | | |
| Surface layer protein [Lactobacillus kefiranofaciens ZW3] | 243 | 5e-76 | AEG41496 |
| Lactobacillus kefiranofaciens subsp. kefiranofaciens DSM 5016 = JCM 6985 [firmicutes ] | | | |
| surface layer protein [Lactobacillus kefiranofaciens subsp. kefiranofaciens DSM 5016 = JCM 6985] | 243 | 5e-76 | KRM20707 |
| Lactobacillus amylovorus [firmicutes ] | | | |
| S-layer protein [Lactobacillus amylovorus] | 242 | 6e-76 | WP_056939632 |
| S-layer protein [Lactobacillus amylovorus] | 236 | 5e-73 | WP_013641412 |
| s-layer protein [Lactobacillus amylovorus] | 236 | 5e-73 | ADZ06361 |
| hypothetical protein [Lactobacillus amylovorus] | 233 | 3e-72 | WP_056985907 |
| S-layer protein [Lactobacillus amylovorus] | 232 | 2e-71 | WP_014565525 |
| S-layer protein [Lactobacillus amylovorus] | 231 | 3e-71 | WP_136860723 |
| S-layer protein [Lactobacillus amylovorus] | 231 | 3e-71 | TJY03618 |
| S-layer protein [Lactobacillus amylovorus] | 221 | 1e-67 | AUX15215 |
| Lactobacillus amylovorus DSM 20531 [firmicutes ] | | | |
| S-layer protein [Lactobacillus amylovorus DSM 20531] | 242 | 6e-76 | ATO53009 |
| surface layer protein [Lactobacillus amylovorus DSM 20531] | 242 | 6e-76 | KRK44657 |
| Lactobacillus helveticus CIRM-BIA 953 [firmicutes ] | | | |
| Surface layer protein [Lactobacillus helveticus CIRM-BIA 953] | 241 | 2e-75 | CDI42150 |

| Description | Score | E value | Accession |
|---|---|---|---|
| **Surface layer protein [Lactobacillus helveticus CIRM-BIA 953]** | 241 | 2e-75 | CDI42266 |
| Lactobacillus gallinarum [firmicutes ] | | | |
| **S-layer protein [Lactobacillus gallinarum]** | 236 | 2e-73 | WP_056945549 |
| **S-layer protein [Lactobacillus gallinarum]** | 234 | 9e-73 | WP_060471252 |
| **S-layer protein [Lactobacillus gallinarum]** | 234 | 9e-73 | ALJ22841 |
| Lactobacillus gallinarum DSM 10532 = JCM 2011 [firmicutes ] | | | |
| **lgsi [Lactobacillus gallinarum DSM 10532 = JCM 2011]** | 236 | 2e-73 | KRL21355 |
| Lactobacillus helveticus H10 [firmicutes ] | | | |
| **Surface layer protein [Lactobacillus helveticus H10]** | 236 | 3e-73 | ADX69414 |
| Lactobacillus crispatus [firmicutes ] | | | |
| **S-layer protein [Lactobacillus crispatus]** | 233 | 3e-72 | WP_123811675 |
| **S-layer protein [Lactobacillus crispatus]** | 232 | 1e-71 | WP_068812872 |
| **SlpB [Lactobacillus crispatus]** | 232 | 1e-71 | ABI49168 |
| **S-layer protein [Lactobacillus crispatus]** | 232 | 1e-71 | PLT12247 |
| **S-layer protein [Lactobacillus crispatus]** | 232 | 1e-71 | PLT14120 |
| Lactobacillus amylovorus DSM 16698 [firmicutes ] | | | |
| **surface layer protein [Lactobacillus amylovorus DSM 16698]** | 233 | 3e-72 | KRN84503 |
| Lactobacillus amylovorus GRL1118 [firmicutes ] | | | |
| **s-layer protein [Lactobacillus amylovorus GRL1118]** | 232 | 2e-71 | AEA31169 |
| Lactobacillus helveticus DPC 4571 [firmicutes ] | | | |
| **Surface layer protein [Lactobacillus helveticus DPC 4571]** | 224 | 1e-68 | ABX26439 |

## Taxonomy

○

| Taxonomy | Number of hits | Number of Organisms | Description |
|---|---|---|---|
| Lactobacillus | 126 | 22 | |
| . Lactobacillus acidophilus | 31 | 5 | **Lactobacillus acidophilus hits** |
| .. Lactobacillus acidophilus NCFM | 5 | 1 | **Lactobacillus acidophilus NCFM hits** |
| .. Lactobacillus acidophilus La-14 | 2 | 1 | **Lactobacillus acidophilus La-14 hits** |
| .. Lactobacillus acidophilus DSM 20242 | 2 | 1 | **Lactobacillus acidophilus DSM 20242 hits** |
| .. Lactobacillus acidophilus CIRM-BIA 442 | 2 | 1 | **Lactobacillus acidophilus CIRM-BIA 442 hits** |
| . Lactobacillus helveticus | 50 | 7 | **Lactobacillus helveticus hits** |
| .. Lactobacillus helveticus DSM 20075 = CGMCC 1.1877 | 3 | 1 | **Lactobacillus helveticus DSM 20075 = CGMCC 1.1877 hits** |
| .. Lactobacillus helveticus CIRM-BIA 101 | 1 | 1 | **Lactobacillus helveticus CIRM-BIA 101 hits** |
| .. Lactobacillus helveticus CIRM-BIA 104 | 1 | 1 | **Lactobacillus helveticus CIRM-BIA 104 hits** |
| .. Lactobacillus helveticus CIRM-BIA 953 | 2 | 1 | **Lactobacillus helveticus CIRM-BIA 953 hits** |
| .. Lactobacillus helveticus H10 | 1 | 1 | **Lactobacillus helveticus H10 hits** |
| .. Lactobacillus helveticus DPC 4571 | 1 | 1 | **Lactobacillus helveticus DPC 4571 hits** |
| . Lactobacillus kefiranofaciens | 2 | 3 | **Lactobacillus kefiranofaciens hits** |
| .. Lactobacillus kefiranofaciens ZW3 | 1 | 1 | **Lactobacillus kefiranofaciens ZW3 hits** |
| .. Lactobacillus kefiranofaciens subsp. kefiranofaciens DSM 5016 = JCM 6985 | 1 | 1 | **Lactobacillus kefiranofaciens subsp. kefiranofaciens DSM 5016 = JCM 6985 hits** |
| . Lactobacillus amylovorus | 8 | 4 | **Lactobacillus amylovorus hits** |
| .. Lactobacillus amylovorus DSM 20531 | 2 | 1 | **Lactobacillus amylovorus DSM 20531 hits** |
| .. Lactobacillus amylovorus DSM 16698 | 1 | 1 | **Lactobacillus amylovorus DSM 16698 hits** |
| .. Lactobacillus amylovorus GRL1118 | 1 | 1 | **Lactobacillus amylovorus GRL1118 hits** |
| . Lactobacillus gallinarum | 3 | 2 | **Lactobacillus gallinarum hits** |
| .. Lactobacillus gallinarum DSM 10532 = JCM 2011 | 1 | 1 | **Lactobacillus gallinarum DSM 10532 = JCM 2011 hits** |
| . Lactobacillus crispatus | 5 | 1 | **Lactobacillus crispatus hits** |

Top
